# Sensitive and immunogen-specific serological detection of *Rodentibacter pneumotropicus* infections in mice

**DOI:** 10.1186/s12866-019-1417-7

**Published:** 2019-02-18

**Authors:** Felix Fingas, Daniela Volke, Rayk Hassert, Juliane Fornefett, Sophie Funk, Christoph Georg Baums, Ralf Hoffmann

**Affiliations:** 1 Institute of Bioanalytical Chemistry, Faculty of Chemistry and Mineralogy, Universität Leipzig, Leipzig, Germany; 2 GVG Diagnostics GmbH, Leipzig, Germany; 3 Center for Biotechnology and Biomedicine, Universität Leipzig, Leipzig, Germany; 4 Institute for Bacteriology and Mycology, Faculty of Veterinary Medicine, Universität Leipzig, Leipzig, Germany; 5 Biotechnologisch-Biomedizinisches Zentrum, Universität Leipzig, Deutscher Platz 5, 04103 Leipzig, Germany

**Keywords:** ELISA, CARLO-1, *Rodentibacter pneumotropicus*, *Rodentibacter heylii*, *Pasteurella pneumotropica*, FELASA, Health monitoring, Antigen, Two-dimensional gel electrophoresis

## Abstract

**Background:**

*Rodentibacter* (*R*.) *pneumotropicus* colonizes the respiratory and urogenital tracts of laboratory mice with a reported moderate serological prevalence from 4 to 13%. Thus, regular tests to identify this pathogen in mice are recommended for animal facilities. However, a recent study indicated that current serological assays are partly insensitive, as C57BL/6 and BALB/c mice infected with *R. pneumotropicus* were incorrectly screened as seronegative.

**Results:**

Here, we report a systematic analysis of protein and lipopolysaccharides antigens by immunoblot and ELISA that allowed establishing a sensitive test system able to differentiate between *R. pneumotropicus* and the closely related species *R. heylii*. Furthermore, the main immunogen, designated as ‘characteristic antigen for *Rodentibacter* of laboratory origin 1’ (CARLO-1), was identified by two-dimensional gel electrophoresis followed by immunoblot and tandem mass spectrometry in a preparation of outer membrane proteins. An indirect ELISA relying on the recombinantly expressed protein provided high sensitivity, specificity, and selectivity. The corresponding *carlo1* gene was highly conserved (> 97%) among 21 isolates of *R. pneumotropicus* and *R. heylii*.

**Conclusion:**

The newly identified protein CARLO-1 is well suited for the sensitive and specific serological detection of *Rodentibacter* infections in mice. Indirect differentiation of *R. pneumotropicus* and *R. heylii* infections may be possible using an ELISA based on a whole-cell antigen preparation. All four established ELISA systems using a whole-cell preparation, lipopolysaccharides, outer-membrane proteins and protein CARLO-1 as antigen, respectively, outperformed a commercial ELISA in terms of sensitivity.

**Electronic supplementary material:**

The online version of this article (10.1186/s12866-019-1417-7) contains supplementary material, which is available to authorized users.

## Background

*Rodentibacter* (*R*.) *pneumotropicus* [*Pasteurella pneumotropica* biotype Jawetz] [1] is a Gram-negative, rod-shaped bacterium of the *Pasteurellaceae* family that frequently colonizes the respiratory and urogenital tracts of laboratory mice and rats. The infection is mostly described as asymptomatic in immunocompetent mice [2], whereas immunodeficient and other genetically modified mice show mild to lethal disease with suppurative lesions in various organs [3]. However, we recently reported high morbidity and mortality in C57BL/6 and BALB/c mice upon infection with a strain isolated from a research facility indicating that the pathology of some *R. pneumotropicus* strains may have been underestimated [4]. Although the pathogen’s environmental stability is very low [5] and transmission mainly depends on direct contact [2], moderate to high prevalence rates of 4 and 13% are reported for laboratory mice in Europe and North America, respectively [6]. Hence, current guidelines of the Federation for Laboratory Animal Science Associations (FELASA) recommend quarterly testing to examine *R. pneumotropicus* infections in mice colonies [7].

The former *Pasteurella* (*P*.) *pneumotropica* biotypes Jawetz and Heyl were recently reclassified into separate species of the genus *Rodentibacter*, i.e., *R. pneumotropicus* and *R. heylii*, respectively [1]. Consequently, sensitive, specific and selective detection assays are required for both species. Current assays rely on direct differentiation based on color, morphological, and biochemical aspects of colonies [1], high-resolution melting curve analysis [8], and DNA amplification using polymerase chain reaction (PCR) [9–11]. Indirect *R. pneumotropicus*- or *R. heylii*-specific serological assays, such as enzyme-linked immunosorbent assay (ELISA), have not been described to the best of our knowledge. Currently, applied ELISA rely on inactivated whole cells detecting both *P. pneumotropica* biotypes Jawetz and Heyl [12, 13]. Manning et al. reported a sensitive detection of *P. pneumotropica* antibodies based on a preparation of uncharacterized cell wall proteins, which were not examined for their specificity to Jawetz and Heyl isolates [14]. ELISA specificity among various members of *Pasteurellaceae* is linked to lipopolysaccharides (LPS), but properties differentiating Jawetz and Heyl species have also not been studied [15]. Vaccination studies identified repeats-in-toxin (RTX) exoproteins and outer membrane protein P6 as protective immunogens [16–18]. Here, we describe a systematic identification of immunogens used to develop a sensitive ELISA detecting specifically *R. pneumotropicus* and *R. heylii* together or only *R. pneumotropicus* without cross-reactivity towards other FELASA-listed bacteria.

## Results

### Confirmation of seroconversion

**Fig. 1:**
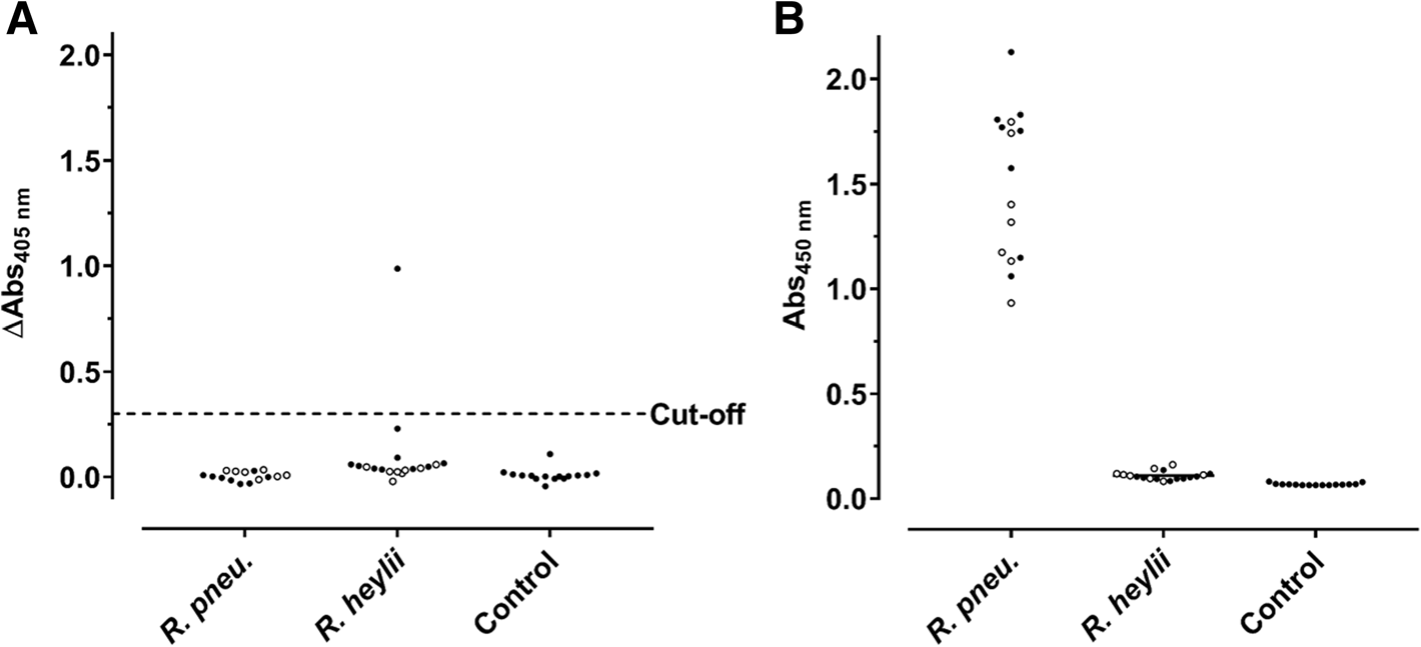
Commercial (**a**) and WCA-based ELISA (**b**) results of sera obtained from C57BL/6 (full circles) and BALB/c mice (open circles) infected with *R. pneumotropicus* or *R. heylii* and uninfected animals. Test specific cut-off value for the commercial ELISA (0.3) is indicated as a dashed line. Similar results of a comparable WCA-ELISA for the *R. pneumotropicus* infected mice and respective controls shown in (**b**) have been published previously [4]

Previously, we reported that immunocompetent C57BL/6 and BALB/c mice intranasally challenged with a *R. pneumotropicus* strain, recently isolated from a laboratory mouse of a German research facility, experienced high rates of morbidity and mortality [4]. Surprisingly, a commercial *Pasteurella pneumotropica* ELISA used for routine health monitoring did not detect *Rodentibacter*-specific antibodies in any of the 15 sera obtained 28 days post infection (dpi; Fig. 1a), although the pathogen was isolated from various organs. Furthermore, this ELISA identified only one of 18 sera from mice experimentally infected with *R. heylii*, providing an overall sensitivity of only 3% (1/33) for both *Rodentibacter* species. Thus, an indirect ELISA using a whole-cell antigen (WCA) extract of the *R. pneumotropicus* strain used in the infection model was established (Fig. 1b), which correctly identified the *R. pneumotropicus*-infected mice with absorbance values ranging from 0.9 to 2.1. The mean absorbance of 1.6 ± 0.4 obtained for C57BL/6 (*n* = 8) was slightly higher than for BALB/c mice (1.4 ± 0.3, *n* = 7) compared to 0.11 ± 0.02 for *R. heylii*-infected mice (*n* = 18) and 0.07 ± 0.005 for uninfected SPF mice (*n* = 15, control). Thus, the WCA-based ELISA was specific and sensitive (15/15) for *R. pneumotropicus* infections.

**Fig. 2:**
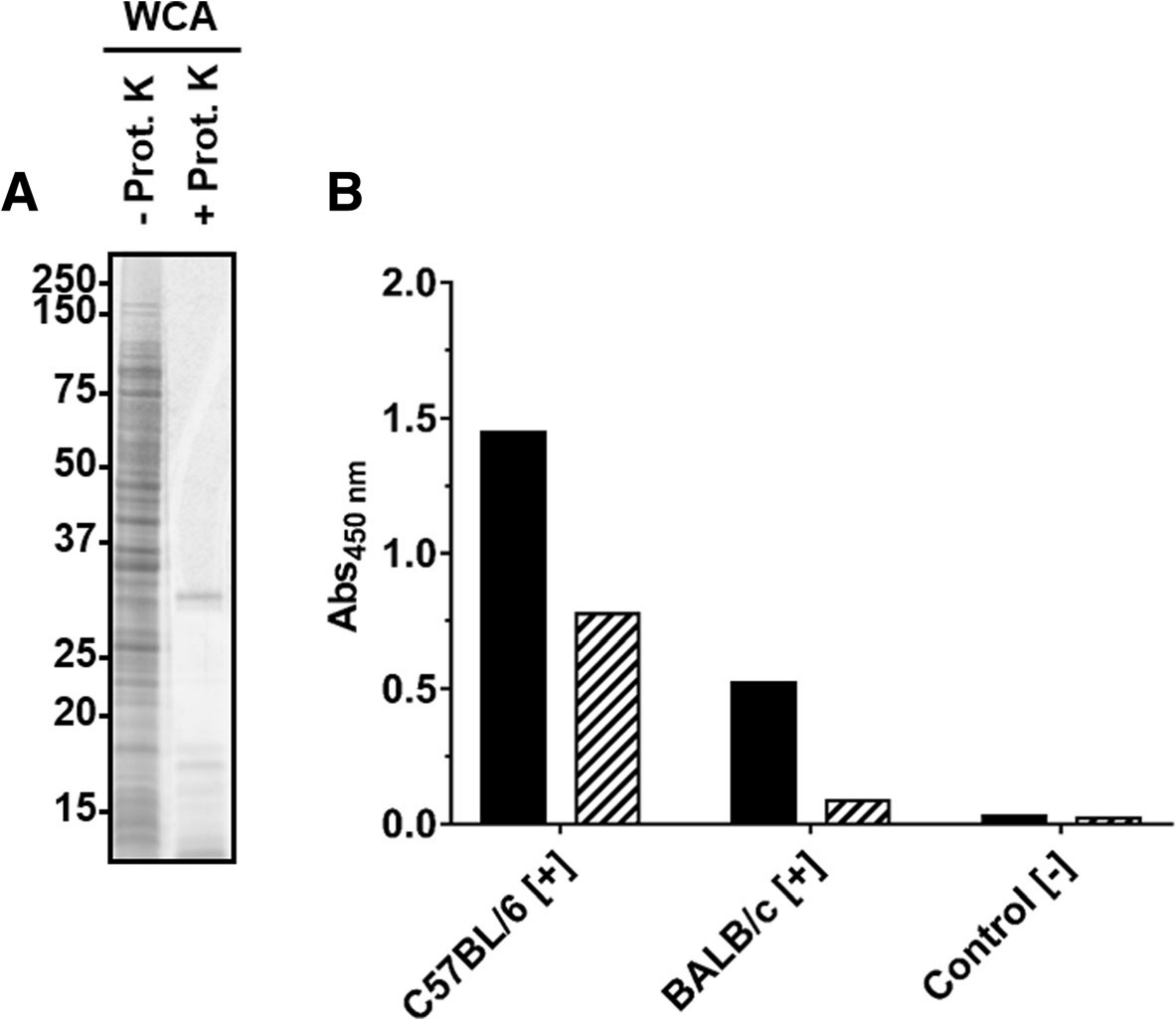
SDS-PAGE (**a**) and ELISA (**b**) of a WCA preparation incubated (56 °C, overnight) in the absence or presence of proteinase K. **a**) Oriole stain of a WCA preparation obtained from *R. pneumotropicus* strain JF4Ni separated by SDS-PAGE before (−) and after (+) proteinase K digestion. **b**) Indirect ELISA using WCA (black) and digested WCA (hatched) probed with sera of experimentally infected C57BL/6 and BALB/c mice and a control serum

As the WCA-ELISA confirmed the seroconversion, the immunogenic components present in the cell extract were further characterized. When proteins present in the WCA preparation were digested with proteinase K (Fig. 2a), as confirmed by SDS-PAGE (30 kDa band corresponds to Protease K), the remaining material was still recognized by sera obtained from both mouse strains in ELISA (Fig. 2b), but with significantly lower readouts. This clearly indicated that the immune system of C57BL/6 and BALB/c mice recognized both proteins and non-proteinogenic components present in the WCA preparation.

### Identification of immunogenic determinants of *R. pneumotropicus*

**Fig. 3:**
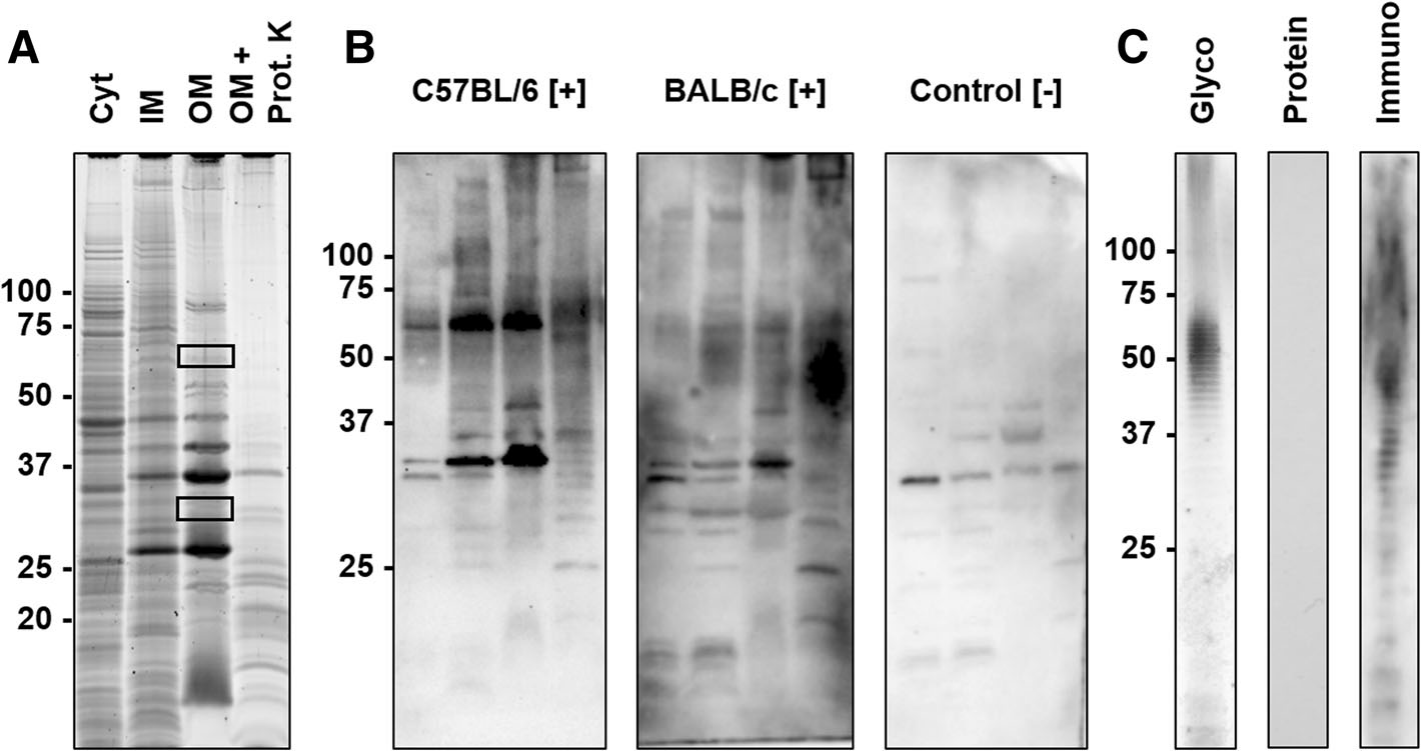
SDS-PAGE and corresponding blots of protein and LPS preparations. **a**) SDS-PAGE of cytosolic (Cyt), sodium *N*-laurylsarcosinate (SLS)-soluble membrane (inner membrane, IM), SLS-insoluble membrane (outer membrane, OM) preparations, and the OM preparation digested with Proteinase K (OM + Prot. K). Proteins were stained with Oriole. Gel areas cut out to identify the proteins by mass spectrometry (MS) are indicated by rectangles. **b**) Immunoblots probed with sera obtained from C57BL/6 and BALB/c mice experimentally infected with *R. pneumotropicus* ([+]) and an uninfected BALB/c mouse (control [−]) **c**) SDS-PAGE of the LPS preparation stained with Pro-Q Emerald (Glyco) or Coomassie (Protein) and the corresponding immunoblot using serum of an infected C57BL/6 mouse (Immuno). Molecular weights (kDa) of the marker proteins are indicated left

Thus, cellular protein fractions and LPS were prepared by sarcosine differential solubility and phenol extraction, respectively, separated by SDS-PAGE and probed with sera of experimentally infected mice in immunoblots (Fig. 3).

**Fig. 4:**
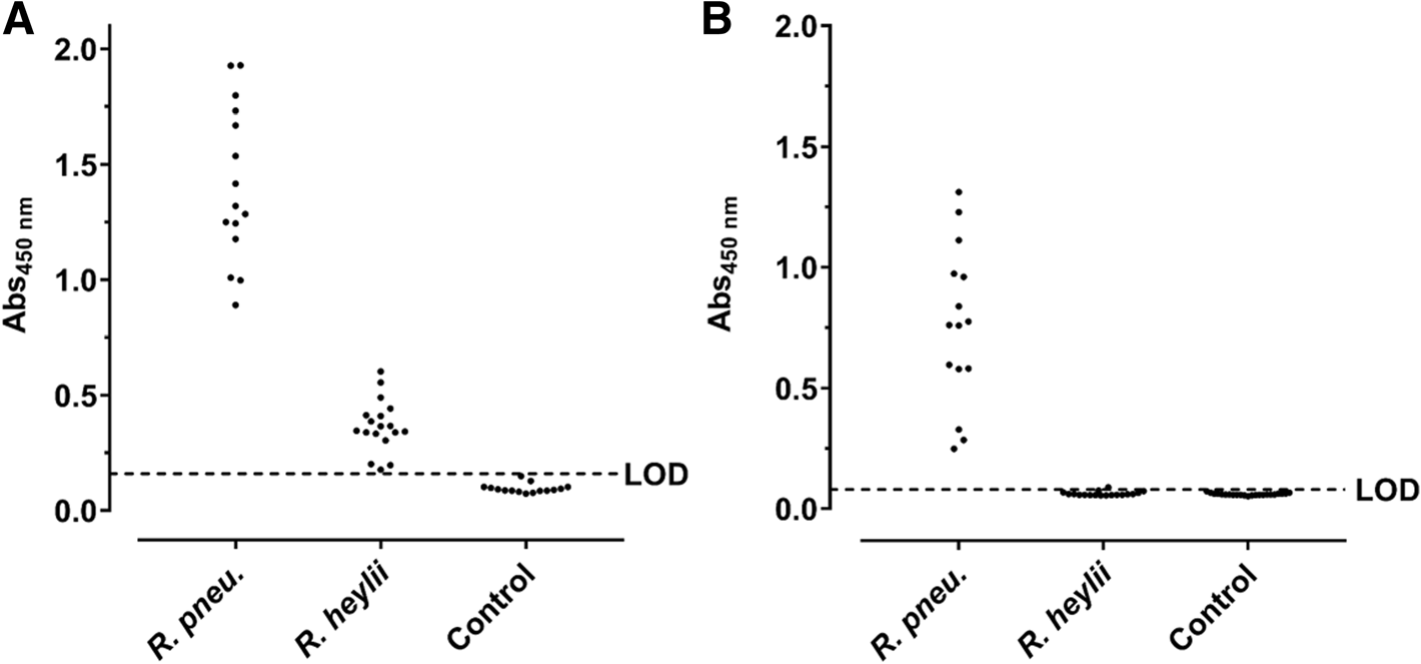
Indirect ELISA based on OM (**a**) and LPS preparations (**b**) probed with sera from C57BL/6 and BALB/c mice experimentally infected with *R. pneumotropicus* or *R. heylii* and uninfected SPF mice (Control). The LODs of OM- (0.16) and LPS-ELISA (0.08) based on the absorbance of control sera are indicated as dashed lines. No differences were observed between the used mouse strains

The protein preparations differed in the band patterns with the number of visible bands decreasing from the Cyt fraction to IM and OM preparations (Fig. 3a). When the OM preparation was digested with proteinase K, most dominant proteins disappeared except for a faint band at an apparent molecular weight of ~ 35 kDa and some weak bands in the lower part of the lane (< 25 kDa). A blot probed with sera of infected C57BL/6 and BALB/c mice displayed a dominant band at ~ 33 kDa and the C57BL/6 serum an additional band at ~ 60 kDa. Both bands also showed the highest relative intensities in the OM preparation. A negative serum stained two bands of low intensity at 30 and 37 kDa. When the corresponding gel bands were cut out (Fig. 3a, rectangles) and digested with trypsin, more than 25 proteins were identified by tandem mass spectrometry (Additional file 1). Interestingly, the two bands were invisible in the immunoblot of the proteinase K-treated OM fraction. Instead, a series of bands ranging from 30 kDa to the top of the gel appeared that were also visible in the IM and OM fractions but absent in the protein stain. This pattern was similar to the patterns obtained by glyco- and immunostaining of the LPS preparation (Fig. 3c). Remarkably, the OM and LPS preparations probed in an indirect ELISA were recognized by sera obtained from *R. pneumotropicus*-infected mice, but not from uninfected mice (Fig. 4).

The readouts of the OM-based ELISA ranged from 0.89 to 1.93 (mean: 1.41 ± 0.34) and the LPS-based ELISA from 0.25 to 1.31 (mean: of 0.76 ± 0.33). When the OM-ELISA was probed with sera of mice infected with the closely related bacterium *R. heylii*, all absorbance values were above the LOD (mean: 0.37 ± 0.11). In contrast, the LPS-ELISA recognized only one of the 15 sera slightly above the LOD (mean value 0.06 ± 0.01). Thus, *R. pneumotropicus* infections appear to trigger a highly specific immune response against LPS that might be applied for the serological differentiation from the closely related species *R. heylii*. However, LPS can be strain-specific and is for example used in the serotyping of *Pasteurella multocida*. Thus, a serological detection solely based on LPS may miss infections with some *R. pneumotropicus* strains.

### Identification of immunogenic proteins of *R. pneumotropicus*

**Fig. 5:**
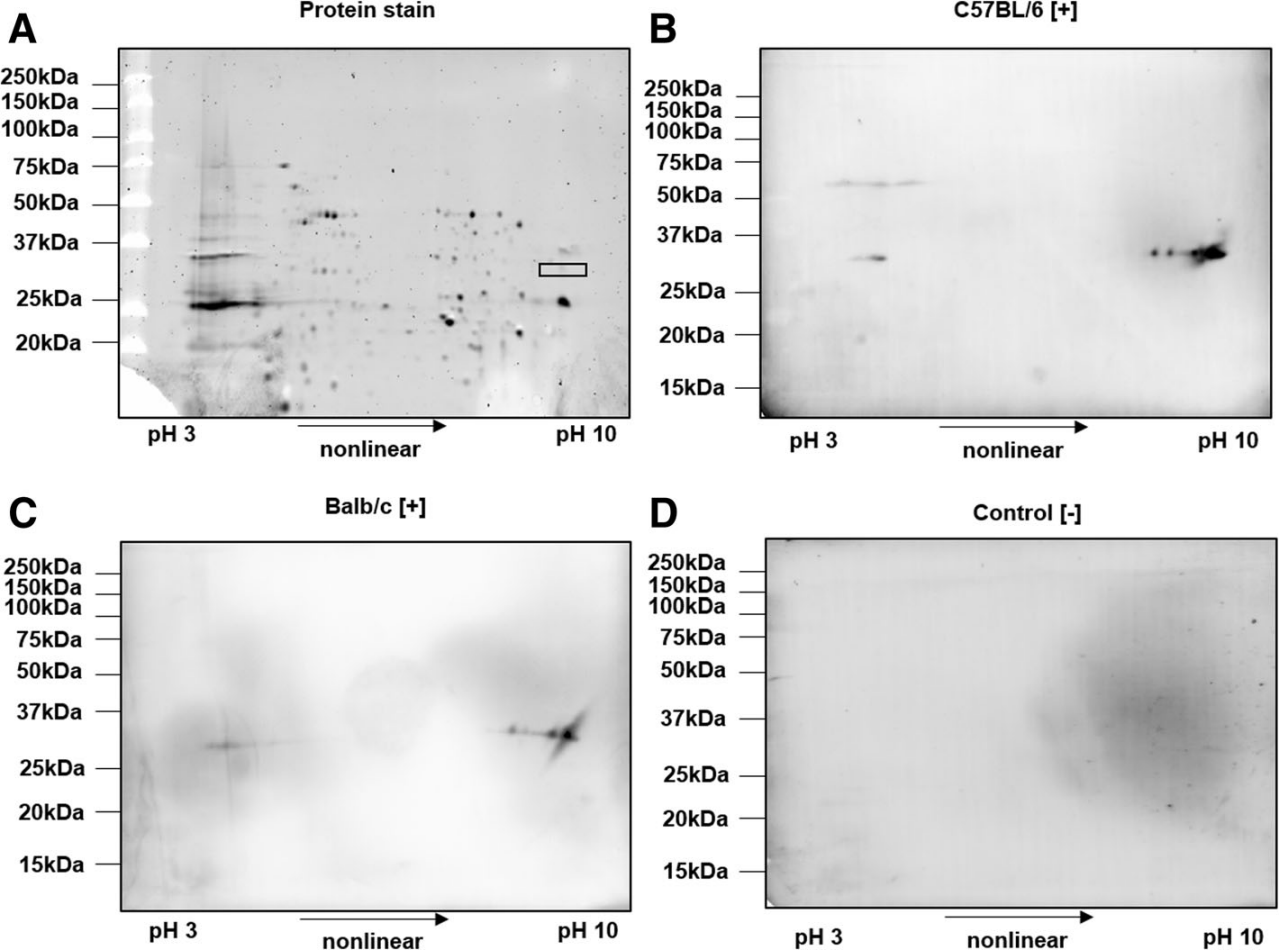
2-DE of SCP of *R. pneumotropicus* stained with Oriole (**a**) and corresponding immunoblots probed with sera obtained from infected C57BL/6 (**b**) and BALB/c mice (**c**) and a non-infected C57BL/6 mouse (control, **d**). Molecular weights of the marker proteins are indicated on the left site. The gel spot area for protein identification by tandem MS is indicated by a rectangle

As the bands recognized by the immune sera after SDS-PAGE contained many proteins, an SDS-lysed cell pellet (SCP) of *R. pneumotropicus* was separated by two-dimensional gel electrophoresis (2-DE) and probed with sera obtained from experimentally infected mice and controls (Fig. 5).

Proteins were well separated in both dimensions and only a few obviously specific signals were observed in the immunoblots probed with sera obtained from infected C57BL/6 and BALB/c mice. The spots detected at an apparent molecular weight of ~ 33 kDa and a pH range from 9 to 10 were identical for sera obtained from both mouse lines. The previously observed signal at 60 kDa for C57BL/6 mice (Fig. 3b, left panel) was also present as a faint series of spots in the acidic part of the gel. Intense spots of immunoblots were matched to a Coomassie stained gel and the corresponding spots were cut and digested with trypsin. Mass spectrometry identified only outer membrane protein A (OmpA). When the OM fraction was applied to 2-DE, similar patterns in immunoblot were observed (data not shown). However, tandem MS identified two additional outer membrane proteins, i.e., long-chain fatty acid transport protein (FadL) and a hypothetical protein (HP), with protein scores above 1000 (Table 1 and Additional file 2).

**Table 1:** Proteins identified in spots after 2-DE corresponding to spots of the immunoblots

Sample	Protein	Accession number	Molecular weight (kDa)	Protein score	Sequence coverage
SCP	OmpA	WP_018357032.1	37.5	4979	61.3%
OM	FadL	WP_018355328.1	48.1	2224	57.1%
OM	HP	WP_018356225.1	34.0	1981	52.9%

### Expression and verification of immunogenic proteins

The three presumed immunogenic proteins (Table 1) of *R. pneumotropicus* were expressed in *E. coli* as recombinant (r) proteins with N-terminal Strep- and C-terminal His-tags. Expression and identity of all three full-length proteins were confirmed by immunoblots after SDS-PAGE using Strep-Tactin or an anti-His-Tag antibody and mass spectrometry after in-gel digestion (Additional files 3, 4, 5). When probed with sera of *R. pneumotropicus*-infected mice, only Strep-rHP-His showed a strong specific signal. The solubilized protein (Additional file 6) was purified by IMAC yielding 0.18 mg pure protein, as indicated by a main band at an apparent molecular weight of 37 kDa (calculated: 34.8 kDa) in SDS-PAGE (Additional file 7), which was also confirmed by tandem mass spectrometry after in-gel digestion (protein score > 23,000, sequence coverage ~ 75%, Additional file 2). The faint band at 30 kDa contained ribosomal protein L2 of *E. coli*.

### Protein-based ELISA

**Fig. 6:**
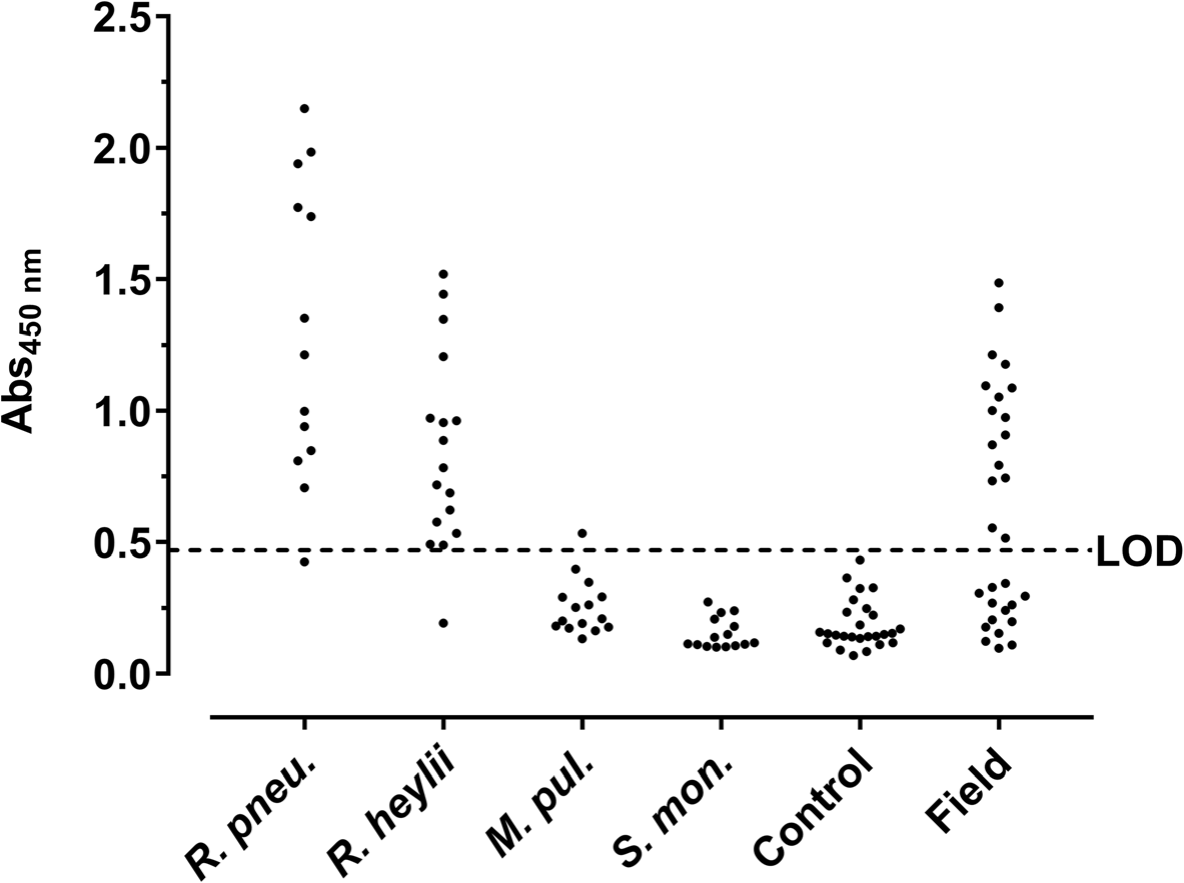
Protein-based ELISA. Strep-rHP-His was coated and probed with sera from mice experimentally infected with *R. pneumotropicus* (*R. pneu.*), *R. heylii*, *M. pulmonis* (*M. pul.*), and *S. moniliformis* (*S. mon.*), sera of uninfected mice (Control), and with field sera. The dashed line indicates the LOD (0.47)

When coated in an indirect ELISA, sera of mice infected with *R. pneumotropicus* generated absorbance values ranging from 0.85 to 2.15 and 0.43 to 1.35 with mean absorbance values of 1.67 ± 0.47 and 0.87 ± 0.31 for C57BL/6 and BALB/c mice, respectively (Fig. 6). Sera of mice infected with *R. heylii* provided absorbance values ranging from 0.53 to 1.52 and 0.19 to 0.96 with mean absorbance values of 1.04 ± 0.36 and 0.64 ± 0.26 for C57BL/6 and BALB/c mice, respectively. This indicates mouse strain specific immune responses upon *Rodentibacter* infections. Sera of uninfected control mice (*n* = 26) resulted in a mean absorbance value of 0.19 ± 0.09 (LOD = 0.47) with all sera tested below 0.43. Thus, 92% (94%) of the sera obtained from mice infected with *R. pneumotropicus* (*R. heylii*) were correctly identified with absorbance values above the LOD. However, the Strep-rHP-His-ELISA could not distinguish infections with the two closely related *Rodentibacter* species. Nevertheless, the antigen was not cross-reactive against serum samples obtained from mice tested positive for other FELASA-listed bacterial pathogens, namely *M. pulmonis* (*n* = 15) and *S. moniliformis* (*n* = 15). Only one of these 30 sera showed an absorbance slightly above the LOD. Thus, *Rodentibacter* infections were detected with high sensitivity and specificity in the probed serum samples using Strep-rHP-His. The diagnostic parameters of the developed ELISA are summarized in Table 2.

**Table 2:** Diagnostic parameters of the indirect Strep-HP-His-ELISA

Diagnostic parameter	Value
Intra-assay CoV	PC: 5.0% (*n* = 42)
	NC: 5.6% (*n* = 42)
Inter-assay CoV	PC: 5.3% (*n* = 5)
	NC: 4.2% (*n* = 5)
LOD	0.47
DSn	93.3% (*n* = 30)
DSp	100% (*n* = 26)
Selectivity	96.7% (*n* = 30)

When serum samples (*n* = 30) obtained from one unit of a German animal facility with acute *R. pneumotropicus* infection, confirmed partially by PCR, cultivation and MALDI-ToF (Additional file 8), were tested, 16 sera were identified as seropositive (Fig. 6, panel ‘field’) compared to only two sera identified positive by a commercial *Pasteurella pneumotropica* ELISA (Additional file 9).

### Conservation of HP on the DNA level in murine *Rodentibacter* isolates

The sequence encoding HP in the genomic DNA was confirmed in *Rodentibacter* isolates classified as *R. pneumotropicus* (*n* = 11) or *R. heylii* (*n* = 10) by PCR [19] (Additional files 10 and 11). Sanger sequencing of the PCR products revealed sequence identities of at least 97% for all tested isolates compared to the *R. pneumotropicus* type strain ATCC35149. Based on the high conservation and the immunogenic character of the identified hypothetical protein HP, it was designated as ‘characteristic antigen for *Rodentibacter* of laboratory origin 1’ and termed CARLO-1.

## Discussion

The recent reclassification of [*Pasteurella pneumotropica*] biotypes Jawetz and Heyl into *R. pneumotropicus* and *R. heylii* [1], respectively, demands a sensitive, specific and selective detection of both pathogens in laboratory mice. So far, the morphological and biochemical differentiation of both species is often inconclusive and, hence, difficult within the manifold *Pasteurellaceae* family [20]. The surprisingly low sensitivity of the applied commercial ELISA observed for confirmed positive sera of *R. pneumotropicus* and *R. heylii* as well as for field sera, may lead to many undetected infections. The WCA-based ELISA established by our group appears to provide a much higher sensitivity and to differentiate defined positive sera of the two closely related *Rodentibacter* species. Apparently, the new test improves previously reported WCA-based ELISA protocols [12, 21]. Generally, the reported prevalence may be influenced by insensitive ELISA assays used in routine health monitoring, as shown here for one commercial assay.

Isolated LPS of *P. multocida* has been shown to be highly protective in mice [22] and was successfully applied as antigen in indirect ELISA and immunoblot for the detection of *P. multocida* infections [23, 24]. However, the LPS isolates were specific for a few serotypes and missed other serotypes, which prevents their general application. Still, they might be useful to confirm a protein-based ELISA.

Recombinantly expressed CARLO-1 provided an indirect ELISA with high sensitivity, specificity, and selectivity. Interestingly, proteins FadL and CARLO-1 were identified in the 2D-gel of the OM fraction, but not from SCP, where only OmpA was identified, which was not recognized in immunoblot after recombinant expression in *E. coli*. Thus, outer membrane protein extracts might be beneficial to identify surface immunogens.

Previous studies identified outer membrane protein P6 [16] and RTX exoproteins, especially PnxIIIA [17, 18], as immunogens in *R. pneumotropicus* Mice intranasally vaccinated with modified recombinant PnxIIIA were protected against *R. pneumotropicus* infections [18] and might thus also be an interesting target for serodiagnosis. However, we recently showed that 18% of the tested *R. pneumotropicus* isolates and 88% of *R. heylii* isolates lack the *pnxIII* gene [4], whereas the *carlo1* gene was present in all 21 analyzed strains of *R. pneumotropicus* and *R. heylii*. Although protein expression and posttranslational modifications were not studied, its presence on the gene level is an important prerequisite for the consistent identification of various *Rodentibacter* strains. By sequencing and in silico analysis of *R. pneumotropicus* and *R. heylii*, Sasaki et al. predicted four genes encoding for YadA-like proteins that were not further characterized [25]. These proteins are often associated with functions in transport, adhesion, and agglutination [26]. The C-terminal sequence of CARLO-1 (residues 238–316) is homologous to the YadA-anchor domain, although the alignment with the N-terminal sequence was not related to any of the sequences reported by Sasaki et al. (data not shown). In silico analysis using the programs PSORTb [27] and LipoP [28] predicted that CARLO-1 is located in the outer membrane, which is supported by its identification in the SLS-insoluble membrane fraction.

## Conclusion

Four ELISA based on various immunogenic components of *R. pneumotropicus* were established that specifically detected antibodies in sera of C57BL/6 and BALB/c mice experimentally infected with *R. pneumotropicus*. The newly identified and recombinantly expressed protein CARLO-1 appeared to be very promising in terms of sensitivity, specificity, and selectivity for the serological detection of *R. pneumotropicus* and *R. heylii* infections. Interestingly, an ELISA based on whole-cell antigen preparation selectively detected infections with *R. pneumotropicus* without cross-reactivity to sera obtained from mice infected with *R. heylii*. Thus, the Strep-rCARLO-1-His-ELISA may be used in routine health monitoring to screen for *Rodentibacter* infections while differentiation may be possible by the WCA-based ELISA.

## Methods

Reagents were obtained from the following manufacturers: Applichem (Darmstadt, Germany): Ethylenediaminetetraacetic acid (EDTA), iodoacetamide, Proteinase K, Tris (hydroxymethyl) aminomethane (Tris base); Bio-Rad Laboratories GmbH (Munich, Germany): Bio-Lyte 3–10 buffer, mineral oil, Oriole™ fluorescent gel stain, Precision Plus Protein™ protein standard (unstained and dual color), Precision protein StrepTactin-HRP conjugate (#350000206), TransBlot Turbo RTA Transfer Kit LF PVDF Mini; Biosolve BV (Valkenswaard, Netherlands): Acetonitrile (HPLC gradient grade) and formic acid (≥99%); Biozym Scientific GmbH (Hessisch Oldendorf, Germany): Advansta blocking buffer, Advansta washing buffer, isopropyl β-D-1-thiogalactopyranoside (IPTG) and WesternBright™ Sirius substrate solution; Carl Roth GmbH (Karlsruhe, Germany): 1,4-Dithiothreitol (DTT, ≥99%), Ampicillin, glycerol (99.5%), brain heart infusion (BHI) broth, hydrochloric acid (37%), lysogeny broth (LB) medium, phenol, phosphate-buffered saline pH 7.4 (PBS), PBS with Tween 20 (PBS-T, pH 7.4), sodium dodecyl sulfate (SDS, ≥99.5%), and urea (≥99.5%); Charles River Laboratories (Sulzfeld, Germany): specific-pathogen-free BALB/c mice used for experimental infection; Elocin-lab GmbH (Oberhausen, Germany): horse serum; GE Healthcare (Fairfield, CT, USA): HisTrap™ HP (1 mL); Honeywell Specialty Chemicals Seelze GmbH (Seelze, Germany): bromophenol blue; Jackson ImmunoResearch Laboratories, Inc. (West Grove, PA, USA): Peroxidase-conjugated AffiniPure goat anti-mouse IgG + IgM (H + L, specific-pathogen-free SPF C57BL/6 mice used for experimental infection; Merck KGaA (Darmstadt, Germany): pET21b vector; NH DyeAGNOSTICS GmbH (Halle, Germany): Immuno Blue HRP Substrate; Oxoid Deutschland GmbH (Wesel, Germany): Columbia agar with sheep blood plus (CBA); Peqlab Biotechnologie GmbH (Erlangen, Germany): agarose (peqGold); Roche Diagnostics International AG (Rotkreuz, Switzerland): DNase I (RNase-free); Serva Electrophoresis GmbH (Heidelberg, Germany): Acrylamide/bis(acrylamide) (30% T, 2.67% C), Albumin bovine (Fraction V, protease-free), CHAPS, TEMED, ammonium persulfate (99%), Coomassie® Brilliant Blue G250, glycine (> 98.5%), protease inhibitor mix B and trypsin (sequencing grade, MS approved); Sigma-Aldrich GmbH (Taufkirchen, Germany): 2-mercaptoethanol (BioUltra), ammonium bicarbonate (≥99.5%), carbonate-bicarbonate buffer, imidazole (≥99.5%), magnesium chloride hexahydrate (MgCl_2_, ≥99%), low-melting agarose, sodium hydroxide, sodium *N*-laurylsarcosinate, thiourea (≥99%) and Triton™ X-100; SurModics Inc. (Eden Prairie, Minnesota, USA): StabilZyme Select®, Assay Diluent (Protein-free); Thermo Fisher Scientific (Waltham, Massachusetts, USA): Albumin standard (2 mg/mL), SuperBlock® (PBS), 6x-His Epitope Tag Antibody (HIS.H8); Seramun Diagnostika GmbH (Heidesee, Germany): TMB substrate solution.

Water was produced in-house using a Purelab Ultra water purification system (resistance > 18.2 MΩ·cm; total organic content < 5 ppb; ELGA LabWater GmbH, Celle, Germany).

### Bacterial strains and culture media

*R. pneumotropicus* strain JF4Ni and *R. heylii* strain SF27GVG were isolated from German research facilities. *R. pneumotropicus* strain ATCC35149 was purchased from ATCC (Manassas, VA, USA). All others strains were kindly provided by Laurentiu Benga (Heinrich-Heine-University, Düsseldorf, Germany). *R. pneumotropicus* and *R. heylii* strains were cultivated overnight at 37 °C on CBA and then transferred to BHI broth supplemented with 10% (*v*/*v*) inactivated and sterile-filtrated horse serum for *R. heylii* strains.

### Serum samples

Sera were obtained from mice experimentally infected with *R. pneumotropicus* [4]. Briefly, specific-pathogen-free (SPF) eight week old female BALB/c (*n* = 16) and C57BL/6 mice (*n* = 16) were intranasally infected with *R. pneumotropicus* strain JF4Ni using a bacterial load of 10^8^ CFU. At 28 dpi, mice were anaesthetized through intraperitoneal application of 100 mg ketamine per kg body weight and 5 mg xylazine per kg body weight, blood was collected by cardiac puncture and the mice finally killed by cervical dislocation. Positive sera of *R. heylii*, *Streptobacillus* (*S.*) *moniliformis*, and *Mycoplasma* (*M.*) *pulmonis* were also obtained from experimental infections ([29] and manuscripts in preparation). Field sera obtained from German animal facilities and tested for different pathogens as recommended by FELASA were provided by GVG Diagnostics including the test results.

### PCR and sequencing

Genomic DNA was isolated from cell pellets of *Rodentibacter* strains using the DNeasy Blood & Tissue Kit and the *Rodentibacter* species identified by PCR [11]. The sequences of *ompA*, *fadL*, and *carlo1* genes were amplified from a mixture of purified genomic DNA (5 μL) as well as forward and reverse primer (2.5 μL each, 10 pmol/L, Eurofins Genomics GmbH, Ebersberg, Table 3), Phusion HF Buffer (10 μL, New England Biolabs Inc., Ipswich, MA, USA), dNTPs (1 μL, 0.1 mol/L), PCR-grade water (28.5 μL), and Phusion High-Fidelity DNA Polymerase (0.5 μL, New England Biolabs Inc.). Amplification was performed on a Mastercycler Nexus (Eppendorf) using an initial denaturation at 95 °C for 2 min followed by 30 cycles (95 °C for 2 min, 55 °C for 30 s, and 72 °C for 55 s) and a final extension (72 °C for 10 min). Samples were stored at 4 °C.

**Table 3:** Sequences of forward (fd) and reverse primers (rev) used to amplify *ompA*, *fadL*, and *carlo1* genes

Target gene	NCBI accession	Primer	Sequence (5′-3′)^a^
*ompA*	WP_018357032.1	OmpA_fd	GAT CAG CCC GGG AAA AAA ACT GCA ATC GCA TT
		OmpA_rev	GAT CAG CTC GAG TTT AGA ACC ATT AAC TGC GA
*fadL*	WP_018355328.1	FadL_fd	GAT CAG CCC GGG AAA AAA TTT AAT CAA TCT TT
		FadL_rev	GAT CAG CTC GAG TTA GAA ACG ATA ATT TAC AT
*carlo1*	WP_018356225.1	CARLO-1_fd	GAT CAG CCC GGG ACA TTG TCA TTA GCC TTA CTT G
		CARLO-1_rev	GAT CAG CTC GAG GCC AGC ATT ATA GCT AAC AC

^a^Underlined restriction sites of *XmaI* (CCC GGG) and *XhoI* (CTCGAG) were used for cloning of amplifications products into pET21b_JF

An aliquot of the PCR product (5 μL) was mixed with DNA Loading Dye (1 μL, Thermo Scientific) and separated on a 1% agarose gel (120 V, 30 min). A GeneRuler 100 bp Plus DNA Ladder (Thermo Scientific) was loaded as standard. The gel image was obtained by UV transillumination. For sequencing, PCR products were purified with the NucleoSpin Gel and PCR Clean-up Kit (Macherey-Nagel, Düren, Germany) according to the manufacturer’s instructions. Briefly, purified PCR product (5 μL, 20–80 ng/μL) were mixed with the forward and reverse primers for *carlo-1* (5 pmol/μL) and sequenced at GATC Biotech (Konstanz, Germany).

### WCA preparation

The WCA was prepared using a previously reported protocol [29]. Briefly, a cell pellet of *R. pneumotropicus* was suspended in B-Per Bacterial Extraction Reagent (4 mL per g pellet, Thermo Scientific), sonicated, and the supernatant dialyzed (8 kDa MW cut-off) against PBS.

### OM preparation

OM proteins were prepared by sarcosine differential solubility [30]. Briefly, an overnight cell culture (0.5 L) was centrifuged (5000 x *g*, 4 °C, 20 min), the cell pellet suspended in Tris-HCl (0.2 mol/L) containing EDTA (10 mmol/L, pH 7.8), and disrupted by three freeze/thaw cycles in liquid nitrogen followed by six cycles of sonication (30 s/cycle, pulse: 1 s, pause: 1 s, 30% amplitude, Vibra-Cell 75,041 with a SM0303 [Sonics & Materials Inc., Newtown, CT, USA]). Protease Inhibitor Mix B (150 μL) was added and the sample centrifuged at 5000 x *g* (10 min, 4 °C). The supernatant was centrifuged at 100,000 x *g* (1 h, 4 °C). The obtained pellet was washed with Tris-HCl (10 mmol/L) containing EDTA (10 mmol/L, pH 7.8; buffer A), suspended in buffer A containing SLS (0.5%, *v*/*v*), incubated (1 h, RT), and centrifuged (100,000 x *g*, 1 h, 4 °C). The supernatant contained the IM proteins. The pellet was washed once with buffer A and suspended in buffer A (overnight, 4 °C) to give the OM fraction. Aliquots were stored at − 80 °C. The protein concentration was determined by micro-Bradford assay.

### Protein digestion

Proteinase K (2 mg) was added to WCA or OM protein solutions (250 μL) and incubated on an orbital shaker (600 rpm, overnight, 56 °C).

### LPS preparation

A cell pellet of *R. pneumotropicus* (140 mg) was suspended in PBS (2 mL), heated (95 °C, 10 min, 600 rpm), and sonicated (3 × 30 s, pulse: 1 s, pause: 1 s, amplitude: 40%, Sonopuls HD2200, MS73 tip [Bandelin]). MgCl_2_ (final 3 mmol/L) and a spatula tip DNase I were added and the solution incubated (30 min, 37 °C). Proteinase K (spatula tip) was added and incubated (600 rpm, 56 °C, overnight). Tris-saturated phenol (2 mL, pH 8.0) was added, mixed, incubated on an orbital shaker (500 rpm, 65 °C, 15 min), and centrifuged (5000 x *g*, 10 min, RT). The upper phase was transferred to a fresh tube and the lower phase was extracted with water (2 mL) and treated as described. After centrifugation, both upper phases were combined and the sample was dialyzed against highly pure water using a Membra-Cel dialysis membrane (3.5 kDa cut-off, 16 mm diameter, Serva Electrophoresis GmbH) until the absorbance of the dialysis water recorded at 260 nm was zero.

### Molecular cloning and protein expression

PCR products were cloned into pET21b-JF (Additional file 12), which is a pET21b plasmid variant containing additional DNA sequence encoding an N-terminal Strep-tag II. Introduced restriction sites for *XmaI* and *XhoI* were used for cloning of amplification products using primers outlined in Table 3 resulting in expression of recombinant proteins with an N-terminal Strep-tag and a C-terminal His-tag. Plasmids were transformed to *E. coli* BL21 by electroporation. LB-medium (0.5 L) was inoculated with an overnight culture and bacteria were grown at 37 °C with continuous shaking until an optical density at 600 nm of 0.6 was obtained. Protein expression was induced by addition of IPTG (1 mmol/L). After 4 h, the culture was centrifuged (7000 x *g*, 15 min, 4 °C) and the cell pellet was stored at − 20 °C. Proteins were solubilized and purified as described [31]. Fractions containing the target protein were dialyzed against 8 mol/L urea in PBS, pH 7.4 (10 kDa MW cut-off) and stored at − 80 °C. Protein concentrations were determined on a NanoDrop 2000c spectrophotometer (Thermo Fisher Scientific, USA) against dialysis buffer as blank.

### Micro-Bradford assay

Protein concentrations of whole-cell extracts and OM preparations were determined by a micro-Bradford assay [32]. Briefly, protein solution (5 μL, duplicates) were mixed with Coomassie Brilliant Blue G-250 solution (250 μL, 0.1 g/L in 10% phosphoric acid and 5% aqueous ethanol) in a 96-well microtiter plate and the absorbance was recorded at 595 nm on a SpectraMax Paradigm microtiter plate reader (Molecular Devices, San José, CA, USA). Three two-fold dilution series of bovine serum albumin (1.0 g/L to 62.5 μg/mL) were used as reference standard.

### SDS-page

SDS-PAGE was performed as described [31]. Proteins were stained using colloidal Coomassie Brilliant Blue (CBB) G-250 [33] or Oriole™ fluorescent stain (λ_exc_ = 270 nm, λ_em_ = 604 nm) according to the manufacturer’s protocol. LPS was stained with the Pro-Q Emerald 300 Glycoprotein Gel and Blot Kit according to the manufacturer’s instructions. Images were taken on a ChemiDoc MP CCD camera system (Bio-Rad Laboratories).

### 2-DE

For 2-DE, 300 mg bacterial pellet were washed twice with 100 mM sucrose (*w*/*v*) and then lysed in 1 mL 2% SDS (*w/v*), 10% glycerol (*v*/*v*), 30 mmol/L Tris (*w/v*), pH 6.8 for 5 min at 95 °C and 500 rpm. After incubation in an ultrasonic bath (5 min, RT), the SCP was centrifuged at 10,000 x *g* (5 min, RT) and the protein concentration of the supernatant was determined by micro-Bradford assay. Proteins were labeled using the Smart Protein Layers Kit and T-Rex Protein Labeling Kit (NH DyeAGNOSTICS GmbH, Halle, Germany) using the manufacturer’s instructions with slight modifications. The pH of the OM was adjusted to 8.0 with 0.1 mol/L sodium hydroxide and samples were diluted with SPL reaction buffer (provided with the kit) to obtain a final protein concentration of 0.5 μg/μL. T-Rex dye (0.5 μL) and SPL Smartanalyzer SMA basic blue (S) (0.5 μL) were added to the protein sample (10 μg) and incubated for 25 min on ice. After the incubation, the labeled sample was further diluted with 2D-sample buffer (7 mol/L urea, 2 mol/L thiourea, 4% CHAPS, 60 mmol/L DTT) to obtain a final protein concentration of 0.4 μg/μL.

IPG strips (7 cm, 3–10 non-linear Strips [Serva]) were passively rehydrated gel-side down in 125 μL rehydration buffer (2 mol/L thiourea, 7 mol/L urea, 4% [*w*/*v*] CHAPS, 30 mmol/L DTT, 0.5% Bio-Lyte 3–10 buffer, 0.0005% bromophenol blue [*v*/*w*]) for 6 h at RT. Strips were placed gel-side up in a cup loading focusing tray. For each IPG strip two electrode wicks were soaked with rehydration buffer (20 μL) and placed at the ends of the strip. The sample cup was placed at the anode and the labeled sample added (50 μL, 20 μg protein). IPG strip and cup were covered with 4 mL mineral oil and transferred to a Protean IEF cell (Bio-Rad Laboratories). Focusing was done at 20 °C using a voltage gradient to 150 V within 1 h (current limited to 50 μA per strip) and increasing further to 300 V and 1000 V within one hour each, and finally to 3000 V within 2 h. The high voltage remained until 12,000 Vh were reached in total. Strips were equilibrated in equilibration buffer (2.5 mL; 6 mol/L urea, 2% SDS, 20% glycerol, 50 mmol/L Tris-HCl, pH 8.8) containing DTT (1%, *w/v*) on an orbital shaker (300 rpm). After 15 min (RT), equilibration buffer (2.5 mL) containing iodoacetamide (2.5%, *w/v*) was added. After a 15-min incubation (300 rpm, RT), strips were immersed in running buffer, placed on top of a polyacrylamide gel (T = 13.5%, C = 2.67%), and fixed with low-melting agarose (0.5%, *w/v*) in Tris-buffer (25 mmol/L Tris-HCL, 192 mmol/L glycine, pH 8.3, 0.1% (*w/v*) SDS) containing 0.001% bromophenol blue.

### Immunoblot

Proteins were electroblotted onto a low-fluorescent PVDF membrane using a Trans-Blot Turbo transfer cell and the RTA transfer kit (Bio-Rad Laboratories) for 10 min (25 V, 1.3 A, RT) and processed as described [31]. For detection, the membrane was incubated with Immuno Blue HRP-Substrate (NH DyeAGNOSTICS, Halle, Germany) for 10 min or with WesternBright Sirius HRP-Substrate (Advansta, Menlo Park, CA, USA) for 2 min, washed in Advan washing solution, and the fluorescence or chemoluminescence recorded (ChemiDoc MP CCD camera system, Bio-Rad Laboratories).

### Enzyme-linked immunosorbent assay (ELISA)

Sera were tested using the Mouse *Pasteurella pneumotropica* ELISA Kit (XpressBio, Frederick, MD, USA) according to the manufacturer’s instructions. The absorbance was recorded at 405 nm (Infinite F50 absorbance microplate reader, Tecan, Männedorf, Switzerland) for each well and the absorbance recorded for control wells subtracted (absorbance difference).

Alternatively, Medium Bind microplates (Brand, Wertheim, Germany; 96-well, U-shape) were coated with 400 ng whole-cell antigen in carbonate-bicarbonate buffer (50 mmol/L, pH 9.6) for 45 min at 37 °C. OM- and LPS-ELISA were prepared by coating OM extract (0.4 μg) and LPS preparation (0.5 μL) in PBS (0.1 mL, pH 7.4) overnight at 4 °C. A solution of Strep-rCARLO-1-His (0.1 μg, 5 μL) in PBS (pH 7.4) containing urea (8 mol/L)) were mixed with PBS (95 μL) in the well and coated overnight (4 °C). Wells were washed three times (PBS, 300 μL) using a Columbus Pro ELISA washer (Tecan), blocked with Superblock (300 μL, 30 min, RT), and stored at 4 °C. During all incubations, plates were covered with adhesive foil (SealPlate, Excel Scientific, CA, USA). Wells were incubated with diluted murine serum (1:50 in Assay Diluent; 100 μL, RT). After 45 min, the wells were washed three times with PBS-T (300 μL/well) and conjugate solution was added (100 μL/well, goat anti-mouse-HRP, 1:30,000 in Stabilzyme Select, 30 min, RT). Wells were washed and TMB added (100 μL, 15 min, RT). Finally, the reaction was stopped with sulfuric acid (100 μL per well, 0.5 mol/L) and the absorbance recorded at 450 nm.

### In-gel digestion and NanoRP-HPLC-ESI-QTOF-MS/MS

Gel bands and spots in CBB G-250-stained gels were digested and peptides processed as described [31]. Data were analyzed with the Progenesis QI search engine using the following parameters: database NCBI “*Pasteurella pneumotropica*” (21,931 sequences, downloaded 22.09.2017), precursor and product MHP window (singly protonated peptide mass of the theoretical sequence) was − 1, “number by match for peptide minimum value” was 3, “number peptide for protein minimum value” was 1, “number by match for protein minimum value” was 7, “protein mass maximum atomic mass unit value” was 250,000, false positive rate value was 4, one missed cleavage site, trypsin as “digester reagent”, and methionine oxidation and cysteine carbamidomethylation as variable modifications. The final fragment peptide table generated by the ion accounting output was filtered for proteins from *R. pneumotropicus* and *R. heylii*. Proteins represented by at least three different peptides and identified by “AutoCurate” (> 95% probability) were considered confident.

### ELISA validation and statistical analysis

The limit of detection (LOD) was calculated from the mean absorbance value of sera from uninfected SPF mice plus three times its standard deviation (SD). Diagnostic sensitivity (DSn), diagnostic specificity (DSp), and selectivity were calculated using the following equations: DSn = [TP/(TP + FN)] × 100; DSp = [TN/(TN + FP)] × 100; Selectivity = [TN_F_/(TN_F_ + FP_F_)] × 100; with TP: true positives, TN: true negatives, FP: false positives, FN: false negatives, and F: sera positive for other FELASA-listed pathogens. Repeatability of the indirect ELISA was tested by 42 replicates of positive (PC, anti-His antibody, 1:4000 in StabilZyme Select) and negative controls each (NC, *R. pneumotropicus* [−] serum pool, 1:50 in Assay Diluent). Intermediate precision was tested by five replicates of PC and NC each on five different plates. Calculations followed ISO 5725-2 [34].

### Software

Graph Pad Prism 7.0 (Graph Pad Software, La Jolla, CA, USA) was used for graphical representations and statistical analysis. Linear regression of standard curves of micro-Bradford assays were calculated with Microsoft Excel 2013 (Microsoft, Albuquerque, NM, USA). Spot matching of 2D-gels and 2D-immunoblots was done using Delta2D v4.6 (Decodon GmbH, Greifswald, Germany). Sequencing results were analyzed with DNA Baser v4.36 (Heracle BioSoft, Arges, Romania) and alignment was performed using BLASTn v2.8 [35].

## Additional files

Additional file 1: Proteins identified from the OM fraction in Fig. 3a by tandem MS. Given are the gel segment, protein accession number at NCBI, protein description, protein score, average mass (Da), number of protein-matched products, number of protein-matched peptides, number of digested peptides and sequence coverage (%). (XLSX 11 kb)

Additional file 2: Proteins identified from 2-DE of SCP and OM fraction and verification of Strep-rCARLO-1-His expression by tandem MS. Given are the sample, database, protein accession number at NCBI, protein description, protein score, average mass (Da), sequence coverage (%), number of protein-matched peptides and the peptide sequences. (XLSX 12 kb)

Additional file 3: SDS-PAGE (A) and immunoblots (B) of different preparations obtained during expression of Strep-rFadL-His in *E. coli*. The following samples were loaded: cell extract of *E. coli* before inducing protein expression (1) and bacterial pellets after 1 h, 2 h and 3 h (2–4), and cytoplasm (5) and inclusion bodies (6) prepared from cells 3 h after the expression was induced by IPTG. 7: SLS-insoluble membrane fraction. Proteins were stained with colloidal CBB G-250. M denotes marker proteins with the molecular masses in kDa indicated left. Immunoblots probed with anti-Strep-Tactin-HRP conjugate and sera obtained from C57BL/6 and BALB/c mice experimentally infected with *R. pneumotropicus* or uninfected (control). Arrow indicates the band corresponding to Strep-rFadL-His and selected for confirmation by tandem mass spectrometry. (TIF 354 kb)

Additional file 4: SDS-PAGE (A) and immunoblots (B) of different preparations obtained during expression of Strep-rOmpA-His in *E. coli*. The following samples were loaded: cell extract of *E. coli* before inducing protein expression (1) and bacterial pellets after 1 h, 2 h and 4 h (2–4), and cytoplasm (5) and inclusion bodies (6) prepared from cells 3 h after the expression was induced by IPTG. Proteins were stained with colloidal CBB G-250. M denotes marker proteins with the molecular masses in kDa indicated left. Immunoblots probed with anti-His mAb and sera obtained from C57BL/6 and BALB/c mice experimentally infected with *R. pneumotropicus* or uninfected (control). Arrow indicates the band corresponding to Strep-rFadL-His and selected for confirmation by tandem mass spectrometry. (TIF 324 kb)

Additional file 5: SDS-PAGE (A) and immunoblots (B) of different preparations obtained during expression of Strep-rHP-His in *E. coli*. The following samples were loaded: cell extract of *E. coli* before inducing protein expression (1) and bacterial pellets after 1 h, 2 h and 4 h (2–4), and cytoplasm (5) and inclusion bodies (6) prepared from cells 3 h after the expression was induced by IPTG. 7: SLS-insoluble membrane fraction. Proteins were stained with colloidal CBB G-250. Proteins were stained with colloidal CBB G-250. M denotes marker proteins with the molecular masses in kDa indicated left. Immunoblots probed with anti-His mAb and sera obtained from C57BL/6 and BALB/c mice experimentally infected with *R. pneumotropicus* or uninfected (control). Arrow indicates the band corresponding to Strep-rHP-His and selected for confirmation by tandem mass spectrometry. (TIF 340 kb)

Additional file 6: SDS-PAGE stained with colloidal CBB G-250 (A) and immunoblot probed with anti-Strep-Tactin-HRP conjugate (B) of solubilized Strep-rHP-His from different preparations and fractions. The following samples were loaded: cytoplasm (C), fractions 1 to 6 (S1-S6) obtained during stepwise solubilization of Strep-rHP-His inclusion bodies. M denotes marker proteins with the molecular masses indicated left. (TIF 196 kb)

Additional file 7: Purification of Strep-rHP-His. SDS-PAGE of recombinantly expressed Strep-rHP-His stained with colloidal Coomassie. M denotes marker proteins with the molecular masses indicated left. (TIF 42 kb)

Additional file 8: Results of *Rodentibacter* identification in field mice. Given are the animal number, the cultivation results based on the characteristics of Gram, oxidase, and morphology, the MALDI-ToF results from corresponding spots of *Pasteurellaceae*, the PCR result of corresponding spots of *Pasteurellaceae*, and the results of the Strep-rCARLO-1-His and the commercial *Pasteurella pneumotropica* ELISA, respectively. (XLSX 9 kb)

Additional file 9: Commercial *Pasteurella pneumotropica* ELISA results of sera obtained from a unit of a German animal facility infected with *R. pneumotropicus*. Test specific cut-off value (0.3) is indicated as a dashed line. (TIF 101 kb)

Additional file 10: Differentiation of *Rodentibacter* strains by PCR [11]. Isolates with a characteristic band at 451 bp were identified as *R. pneumotropicus* (A), whereas isolates showing a band at 326 bp were identified as *R. heylii* (B). Control: No DNA was added to the PCR reaction. (TIF 239 kb)

Additional file 11: HP-screening in murine isolates of *R. pneumotropicus* (A) and *R. heylii* (B) by PCR. Control [−]: No template was added to the PCR reaction. 100 bp molecular marker is indicated left. (TIF 165 kb)

Additional file 12: Sequence of pET21b_JF (5406 bp). Nucleotide sequence of vector pET21b_JF encoding Strep-tag II and restrictions sites *XmaI* and *XhoI*. (PDF 116 kb)

## Abbreviations

2-DE: two-dimensional gel electrophoresis
CARLO-1: Characteristic antigen for *Rodentibacter* of laboratory origin 1
CoV: Coefficient of variation
Cyt: Cytosolic fraction
dpi: Days post infection
DSn: Diagnostic sensitivity
DSp: Diagnostic specificity
*E. coli*: *Escherichia coli*
ELISA: Enzyme-linked immunosorbent assay
ESI: Electrospray ionization
FadL: Long-chain fatty acid transport protein
FELASA: Federation of Laboratory Animal Science Associations
HP: Hypothetical protein
IM: SLS-soluble membrane fraction
IMAC: Immobilized metal ion affinity chromatography
LOD: Limit of detection
LPS: Lipopolysaccharides
MS: Mass spectrometry
MW: Molecular weight
NC: Negative control
OM: SLS-insoluble membrane fraction
OmpA: Outer-membrane protein A
*P.*: *Pasteurella*
PC: Positive control
PCR: Polymerase chain reaction
QTOF: Quadrupole time-of-flight
*R.*: *Rodentibacter*
RP-HPLC: Reversed-phase high performance liquid chromatography
RTX: Repeats-in-toxin
*S. mon.*: *Streptobacillus moniliformis*
SCP: SDS-lysed cell pellet
SDS-PAGE: Sodium dodecyl sulfate polyacrylamide gel electrophoresis
SLS: Sodium *N*-laurylsarcosinate
SPF: Specific-pathogen-free
WCA: Whole-cell antigen
