# Supplementary figures and images for: Sensitive and immunogen-specific serological detection of Rodentibacter pneumotropicus infections in mice
Source: BMC Microbiol. 2019 Feb 18;19:43. doi: 10.1186/s12866-019-1417-7 (PMC6380038; doi:10.1186/s12866-019-1417-7)

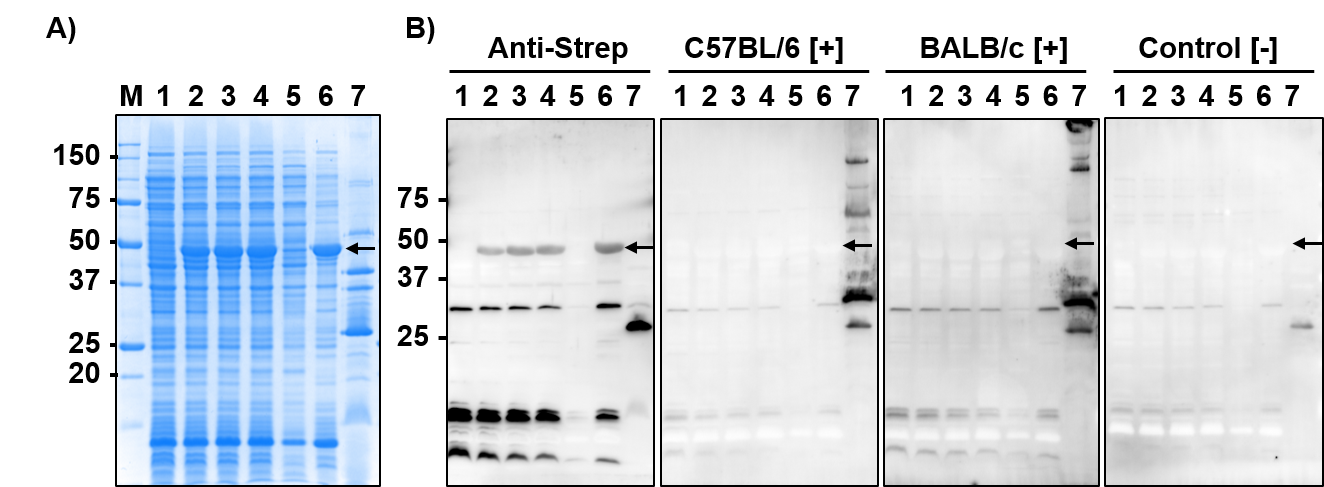

Supplement: Supplementary file 3 — SDS-PAGE (A) and immunoblots (B) of different preparations obtained during expression of Strep-rFadL-His in E. coli. The following samples were loaded: cell extract of E. coli before inducing protein expression (1) and bacterial pellets after 1 h, 2 h and 3 h (2–4), and cytoplasm (5) and inclusion bodies (6) prepared from cells 3 h after the expression was induced by IPTG. 7: SLS-insoluble membrane fraction. Proteins were stained with colloidal CBB G-250. M denotes marker proteins with the molecular masses in kDa indicated left. Immunoblots probed with anti-Strep-Tactin-HRP conjugate and sera obtained from C57BL/6 and BALB/c mice experimentally infected with R. pneumotropicus or uninfected (control). Arrow indicates the band corresponding to Strep-rFadL-His and selected for confirmation by tandem mass spectrometry. (TIF 354 kb) [file 12866_2019_1417_MOESM3_ESM.tif]

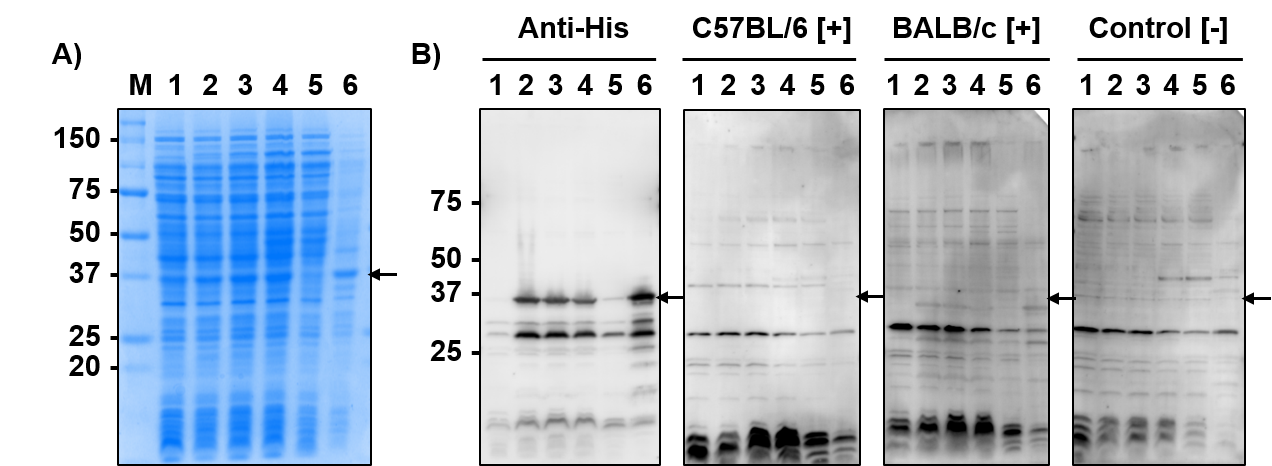

Supplement: Supplementary file 4 — SDS-PAGE (A) and immunoblots (B) of different preparations obtained during expression of Strep-rOmpA-His in E. coli. The following samples were loaded: cell extract of E. coli before inducing protein expression (1) and bacterial pellets after 1 h, 2 h and 4 h (2–4), and cytoplasm (5) and inclusion bodies (6) prepared from cells 3 h after the expression was induced by IPTG. Proteins were stained with colloidal CBB G-250. M denotes marker proteins with the molecular masses in kDa indicated left. Immunoblots probed with anti-His mAb and sera obtained from C57BL/6 and BALB/c mice experimentally infected with R. pneumotropicus or uninfected (control). Arrow indicates the band corresponding to Strep-rFadL-His and selected for confirmation by tandem mass spectrometry. (TIF 324 kb) [file 12866_2019_1417_MOESM4_ESM.tif]

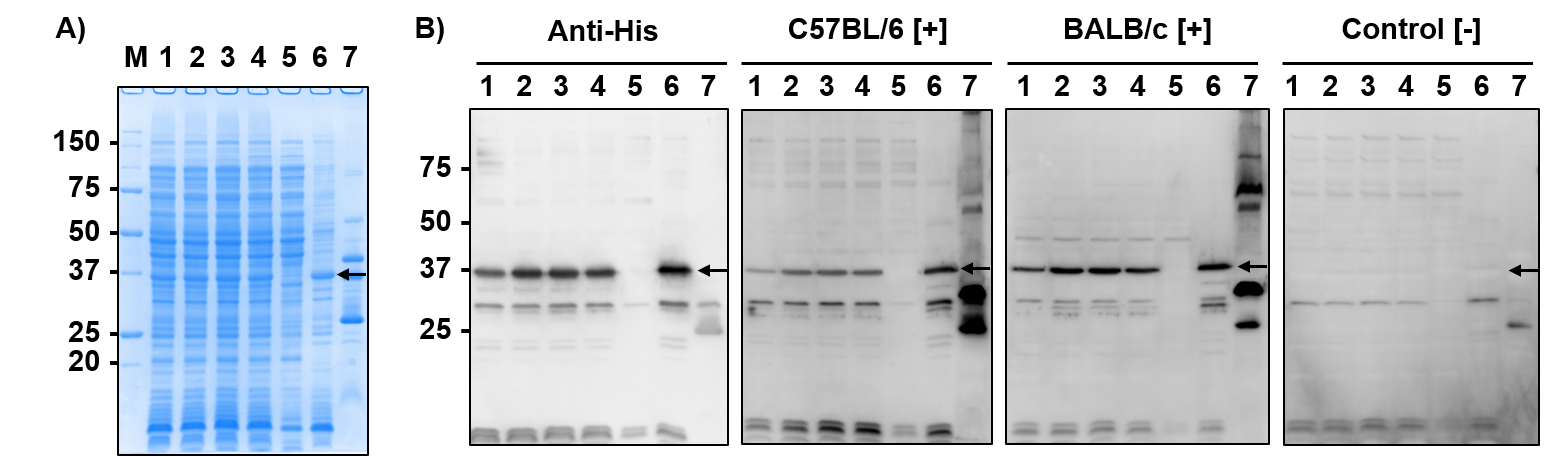

Supplement: Supplementary file 5 — SDS-PAGE (A) and immunoblots (B) of different preparations obtained during expression of Strep-rHP-His in E. coli. The following samples were loaded: cell extract of E. coli before inducing protein expression (1) and bacterial pellets after 1 h, 2 h and 4 h (2–4), and cytoplasm (5) and inclusion bodies (6) prepared from cells 3 h after the expression was induced by IPTG. 7: SLS-insoluble membrane fraction. Proteins were stained with colloidal CBB G-250. Proteins were stained with colloidal CBB G-250. M denotes marker proteins with the molecular masses in kDa indicated left. Immunoblots probed with anti-His mAb and sera obtained from C57BL/6 and BALB/c mice experimentally infected with R. pneumotropicus or uninfected (control). Arrow indicates the band corresponding to Strep-rHP-His and selected for confirmation by tandem mass spectrometry. (TIF 340 kb) [file 12866_2019_1417_MOESM5_ESM.tif]

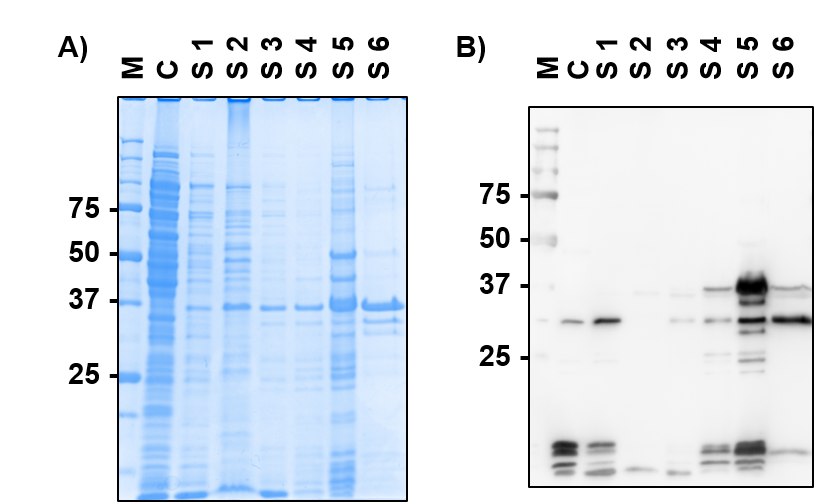

Supplement: Supplementary file 6 — SDS-PAGE stained with colloidal CBB G-250 (A) and immunoblot probed with anti-Strep-Tactin-HRP conjugate (B) of solubilized Strep-rHP-His from different preparations and fractions. The following samples were loaded: cytoplasm (C), fractions 1 to 6 (S1-S6) obtained during stepwise solubilization of Strep-rHP-His inclusion bodies. M denotes marker proteins with the molecular masses indicated left. (TIF 196 kb) [file 12866_2019_1417_MOESM6_ESM.tif]

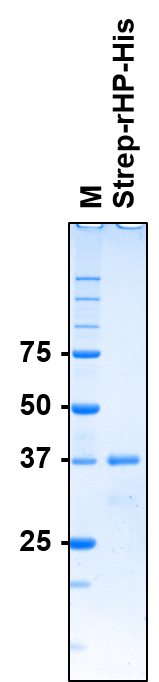

Supplement: Supplementary file 7 — Purification of Strep-rHP-His. SDS-PAGE of recombinantly expressed Strep-rHP-His stained with colloidal Coomassie. M denotes marker proteins with the molecular masses indicated left. (TIF 42 kb) [file 12866_2019_1417_MOESM7_ESM.tif]

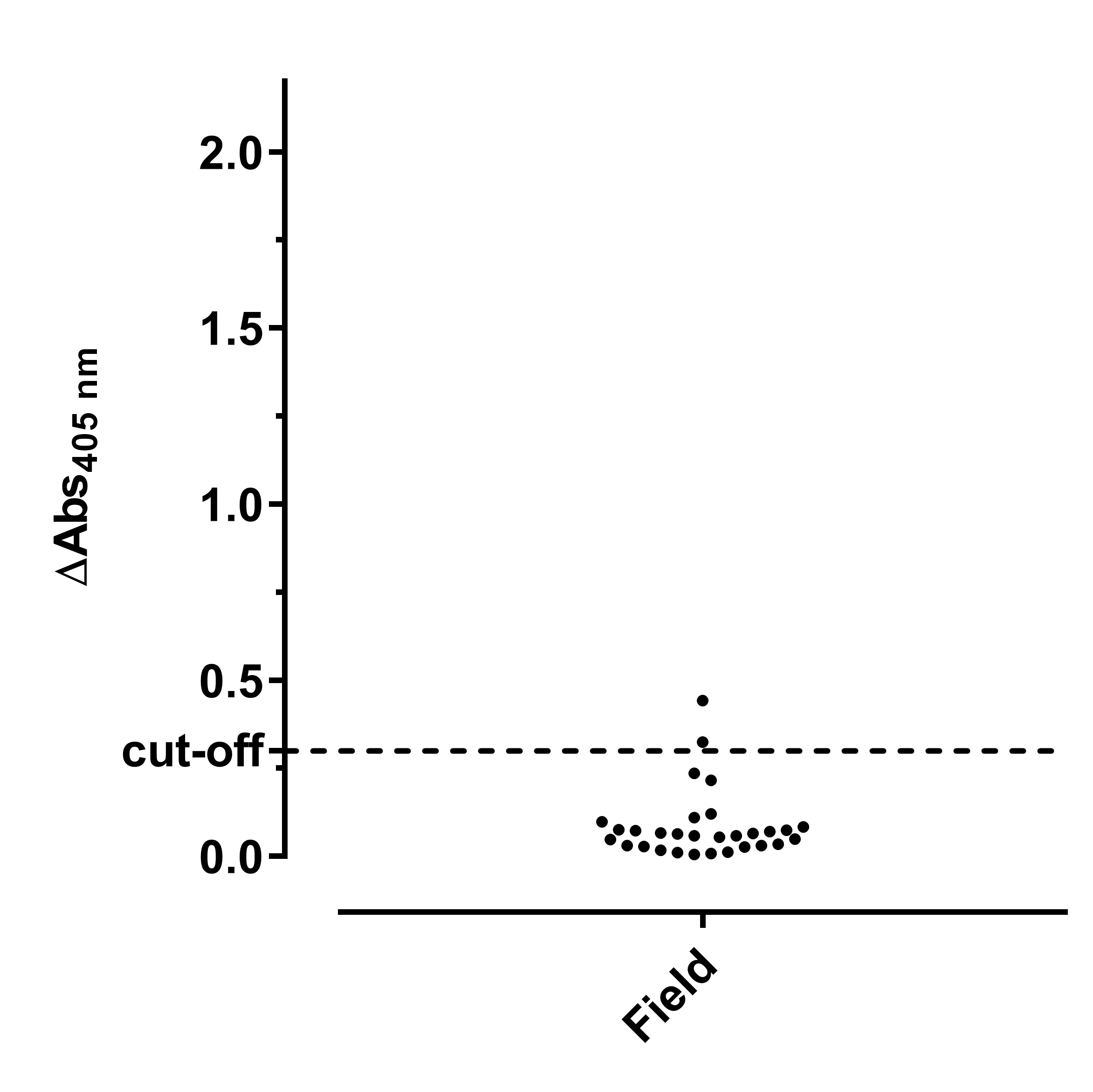

Supplement: Supplementary file 9 — Commercial Pasteurella pneumotropica ELISA results of sera obtained from a unit of a German animal facility infected with R. pneumotropicus. Test specific cut-off value (0.3) is indicated as a dashed line. (TIF 101 kb) [file 12866_2019_1417_MOESM9_ESM.tif]

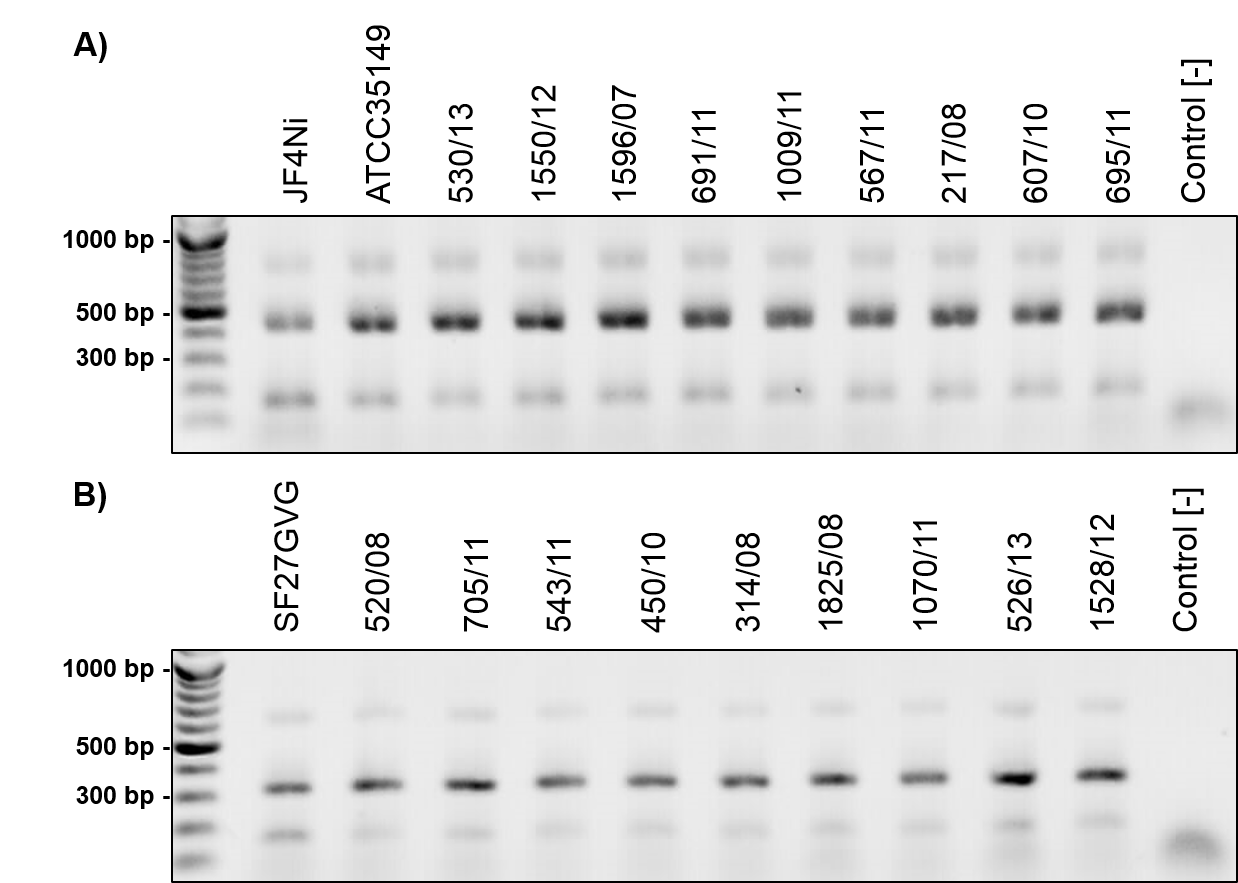

Supplement: Supplementary file 10 — Differentiation of Rodentibacter strains by PCR [11]. Isolates with a characteristic band at 451 bp were identified as R. pneumotropicus (A), whereas isolates showing a band at 326 bp were identified as R. heylii (B). Control: No DNA was added to the PCR reaction. (TIF 239 kb) [file 12866_2019_1417_MOESM10_ESM.tif]

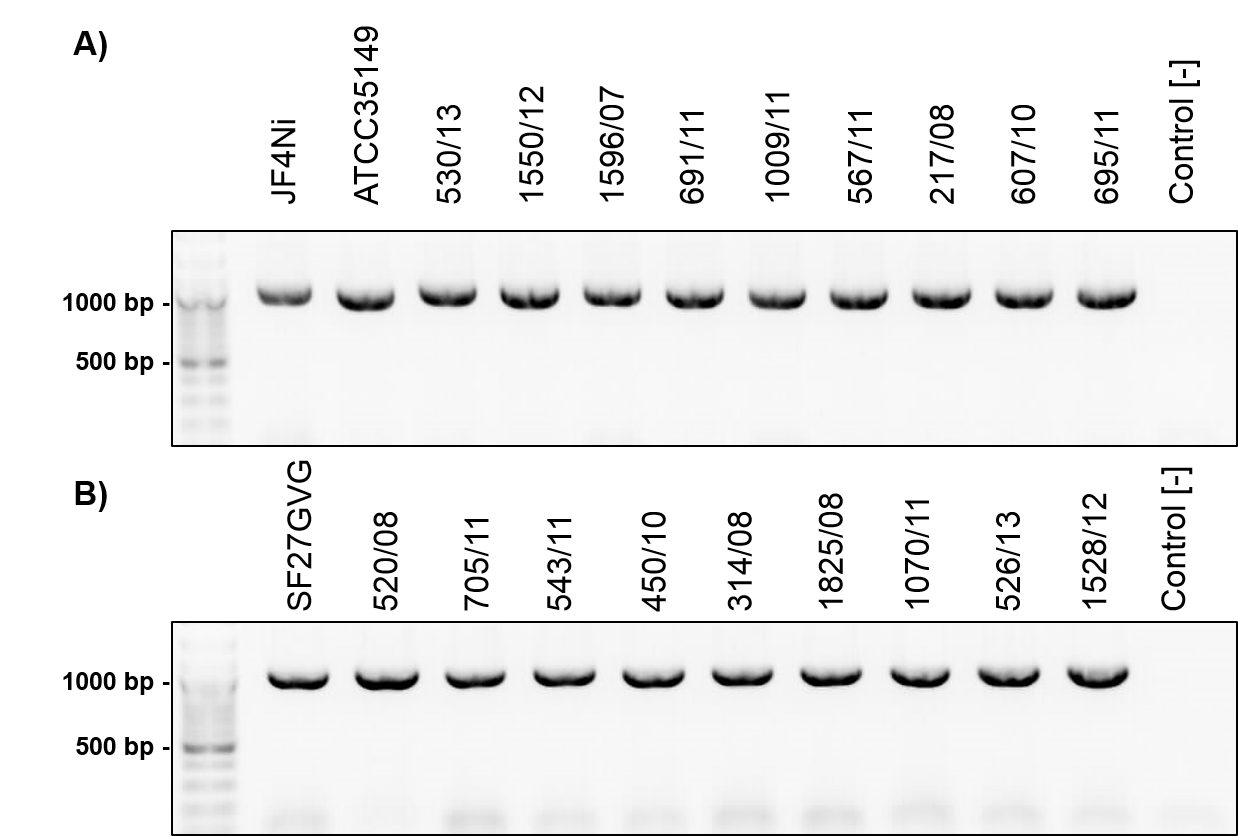

Supplement: Supplementary file 11 — HP-screening in murine isolates of R. pneumotropicus (A) and R. heylii (B) by PCR. Control [−]: No template was added to the PCR reaction. 100 bp molecular marker is indicated left. (TIF 165 kb) [file 12866_2019_1417_MOESM11_ESM.tif]
