# Supplementary material for: Sensitive and immunogen-specific serological detection of Rodentibacter pneumotropicus infections in mice
Source: BMC Microbiol. 2019 Feb 18;19:43. doi: 10.1186/s12866-019-1417-7 (PMC6380038; doi:10.1186/s12866-019-1417-7)
Supplement: Supplementary file 12 — Sequence of pET21b_JF (5406 bp). Nucleotide sequence of vector pET21b_JF encoding Strep-tag II and restrictions sites XmaI and XhoI. (PDF 116 kb) [file 12866_2019_1417_MOESM12_ESM.pdf]

```
Molecule:        pET21b_JF,  5406 bps DNA Circular
File Name:       pet21b_JF.cm5,  dated 09 Aug 2018
Description:     expression of N-term Strep-tag and C-term His-tag
                 recombinant protein
Printed:         1 to 5406 bps (Full)


                                                         XbaI
  1  taatacgact cactataggg gaattgtgag cggataacaa ttcccctcta gaaataattt
     attatgctga gtgatatccc cttaacactc gcctattgtt aaggggagat ctttattaaa

                                  NdeI                            XmaI
 61  tgtttaactt taagaaggag atatacatat gtggagccac ccgcaatttg aaaaacccgg
     acaaattgaa attcttcctc tatatgtata cacctcggtg ggcgttaaac tttttgggcc
                                   >>.........Strep_tag.........>>
                                      m  w  s  h   p  q  f   e  k

              PspXI
              XhoI
121  ggcccagctc gagcaccacc accaccacca ctgagatccg gctgctaaca aagcccgaaa
     ccgggtcgag ctcgtggtgg tggtggtggt gactctaggc cgacgattgt ttcgggcttt
                   >>..... His_tag......>>
                     h  h   h  h  h   h  -

                                   BlpI                    StyI
181  ggaagctgag ttggctgctg ccaccgctga gcaataacta gcataacccc ttggggcctc
     ccttcgactc aaccgacgac ggtggcgact cgttattgat cgtattgggg aaccccggag

241  taaacgggtc ttgaggggtt ttttgctgaa aggaggaact atatccggat tggcgaatgg
     atttgcccag aactccccaa aaaacgactt tcctccttga tataggccta accgcttacc

301  gacgcgccct gtagcggcgc attaagcgcg gcgggtgtgg tggttacgcg cagcgtgacc
     ctgcgcggga catcgccgcg taattcgcgc cgcccacacc accaatgcgc gtcgcactgg

361  gctacacttg ccagcgccct agcgcccgct cctttcgctt tcttcccttc ctttctcgcc
     cgatgtgaac ggtcgcggga tcgcgggcga ggaaagcgaa agaagggaag gaaagagcgg

421  acgttcgccg gctttccccg tcaagctcta aatcgggggc tccctttagg gttccgattt
     tgcaagcggc cgaaaggggc agttcgagat ttagcccccg agggaaatcc caaggctaaa

                                                             DraIII
481  agtgctttac ggcacctcga ccccaaaaaa cttgattagg gtgatggttc acgtagtggg
     tcacgaaatg ccgtggagct ggggtttttt gaactaatcc cactaccaag tgcatcaccc

541  ccatcgccct gatagacggt ttttcgccct ttgacgttgg agtccacgtt ctttaatagt
     ggtagcggga ctatctgcca aaaagcggga aactgcaacc tcaggtgcaa gaaattatca

601  ggactcttgt tccaaactgg aacaacactc aaccctatct cggtctattc ttttgattta
     cctgagaaca aggtttgacc ttgttgtgag ttgggataga gccagataag aaaactaaat

    PsiI
661  taagggattt tgccgatttc ggcctattgg ttaaaaaatg agctgattta acaaaaattt
     attccctaaa acggctaaag ccggataacc aatttttttac tcgactaaat tgtttttaaa

721  aacgcgaatt ttaacaaaat attaacgttt acaatttcag gtggcacttt tcggggaaat
     ttgcgcttaa aattgtttta taattgcaaa tgttaaagtc caccgtgaaa agccccttta

781  gtgcgcggaa cccctatttg tttatttttc taaatacatt caaatatgta tccgctcatg
     cacgcgcctt ggggataaac aaataaaaag atttatgtaa gtttatacat aggcgagtac

841  agacaataac cctgataaat gcttcaataa tattgaaaaa ggaagagtat gagtattcaa
     tctgttattg ggactattta cgaagttatt ataacttttt ccttctcata ctcataagtt
                                                         >>..AmpR....>
                                                            m  s  i  q

901  catttccgtg tcgcccttat tccctttttt gcggcatttt gccttcctgt ttttgctcac
     gtaaaggcac agcgggaata agggaaaaaa cgccgtaaaa cggaaggaca aaaacgagtg
     >.............................AmpR..............................>
       h  f  r   v  a  l   i  p  f  f   a  a  f   c  l  p   v  f  a  h
```

```
 961  ccagaaacgc tggtgaaagt aaaagatgct gaagatcagt tgggtgcacg agtgggttac
      ggtctttgcg accactttca ttttctacga cttctagtca acccacgtgc tcacccaatg
      >..........................AmpR.............................>
        p  e  t   l  v  k   v  k  d  a   e  d  q   l  g  a   r  v  g  y

1021  atcgaactgg atctcaacag cggtaagatc cttgagagtt ttcgccccga agaacgtttt
      tagcttgacc tagagttgtc gccattctag gaactctcaa aagcggggct tcttgcaaaa
      >..........................AmpR.............................>
        i  e  l   d  l  n   s  g  k  i   l  e  s   f  r  p   e  e  r  f

1081  ccaatgatga gcacttttaa agttctgcta tgtggcgcgg tattatcccg tattgacgcc
      ggttactact cgtgaaaatt tcaagacgat acaccgcgcc ataatagggc ataactgcgg
      >..........................AmpR.............................>
        p  m  m   s  t  f   k  v  l  l   c  g  a   v  l  s   r  i  d  a

                                                           ScaI
1141  gggcaagagc aactcggtcg ccgcatacac tattctcaga atgacttggt tgagtactca
      cccgttctcg ttgagccagc ggcgtatgtg ataagagtct tactgaacca actcatgagt
      >..........................AmpR.............................>
        g  q  e   q  l  g   r  r  i  h   y  s  q   n  d  l   v  e  y  s

1201  ccagtcacag aaaagcatct tacggatggc atgacagtaa gagaattatg cagtgctgcc
      ggtcagtgtc ttttcgtaga atgcctaccg tactgtcatt ctcttaatac gtcacgacgg
      >..........................AmpR.............................>
        p  v  t   e  k  h   l  t  d  g   m  t  v   r  e  l   c  s  a  a

                                                 PvuI
1261  ataaccatga gtgataacac tgcggccaac ttacttctga caacgatcgg aggaccgaag
      tattggtact cactattgtg acgccggttg aatgaagact gttgctagcc tcctggcttc
      >..........................AmpR.............................>
        i  t  m   s  d  n   t  a  a  n   l  l  l   t  t  i   g  g  p  k

1321  gagctaaccg cttttttgca caacatgggg gatcatgtaa ctcgccttga tcgttgggaa
      ctcgattggc gaaaaaacgt gttgtacccc ctagtacatt gagcggaact agcaaccctt
      >..........................AmpR.............................>
        e  l  t   a  f  l   h  n  m  g   d  h  v   t  r  l   d  r  w  e

                                                       PstI
1381  ccggagctga atgaagccat accaaacgac gagcgtgaca ccacgatgcc tgcagcaatg
      ggcctcgact tacttcggta tggtttgctg ctcgcactgt ggtgctacgg acgtcgttac
      >..........................AmpR.............................>
        p  e  l   n  e  a   i  p  n  d   e  r  d   t  t  m   p  a  a  m

1441  gcaacaacgt tgcgcaaact attaactggc gaactactta ctctagcttc ccggcaacaa
      cgttgttgca acgcgtttga taattgaccg cttgatgaat gagatcgaag ggccgttgtt
      >..........................AmpR.............................>
        a  t  t   l  r  k   l  l  t  g   e  l  l   t  l  a   s  r  q  q

                                                            BglI
1501  ttaatagact ggatggaggc ggataaagtt gcaggaccac ttctgcgctc ggcccttccg
      aattatctga cctacctccg cctatttcaa cgtcctggtg aagacgcgag ccgggaaggc
      >..........................AmpR.............................>
        l  i  d   w  m  e   a  d  k  v   a  g  p   l  l  r   s  a  l  p

                                                 BsaI
1561  gctggctggt ttattgctga taaatctgga gccggtgagc gtgggtctcg cggtatcatt
      cgaccgacca aataacgact atttagacct cggccactcg cacccagagc gccatagtaa
      >..........................AmpR.............................>
        a  g  w   f  i  a   d  k  s  g   a  g  e   r  g  s   r  g  i  i

1621  gcagcactgg ggccagatgg taagccctcc cgtatcgtag ttatctacac gacggggagt
      cgtcgtgacc ccggtctacc attcgggagg gcatagcatc aatagatgtg ctgcccctca
      >..........................AmpR.............................>
        a  a  l   g  p  d   g  k  p  s   r  i  v   v  i  y   t  t  g  s
```

```
1681  caggcaacta tggatgaacg aaatagacag atcgctgaga taggtgcctc actgattaag
      gtccgttgat acctacttgc tttatctgtc tagcgactct atccacggag tgactaattc
      >..........................AmpR............................>
        q  a  t   m  d  e   r  n  r  q   i  a  e   i  g  a   s  l  i  k

1741  cattggtaac tgtcagacca agtttactca tatatacttt agattgattt aaaacttcat
      gtaaccattg acagtctggt tcaaatgagt atatatgaaa tctaactaaa ttttgaagta
      >.AmpR.>>
        h  w  -

1801  ttttaattta aaaggatcta ggtgaagatc ctttttgata atctcatgac caaaatccct
      aaaattaaat tttcctagat ccacttctag gaaaaactat tagagtactg gttttaggga

1861  taacgtgagt tttcgttcca ctgagcgtca gaccccgtag aaaagatcaa aggatcttct
      attgcactca aaagcaaggt gactcgcagt ctggggcatc ttttctagtt tcctagaaga

1921  tgagatcctt tttttctgcg cgtaatctgc tgcttgcaaa caaaaaaacc accgctacca
      actctaggaa aaaaagacgc gcattagacg acgaacgttt gtttttttgg tggcgatggt

1981  gcggtggttt gtttgccgga tcaagagcta ccaactcttt ttccgaaggt aactggcttc
      cgccaccaaa caaacggcct agttctcgat ggttgagaaa aaggcttcca ttgaccgaag

2041  agcagagcgc agataccaaa tactgtcctt ctagtgtagc cgtagttagg ccaccacttc
      tcgtctcgcg tctatggttt atgacaggaa gatcacatcg gcatcaatcc ggtggtgaag

                                                         AlwNI
2101  aagaactctg tagcaccgcc tacatacctc gctctgctaa tcctgttacc agtggctgct
      ttcttgagac atcgtggcgg atgtatggag cgagacgatt aggacaatgg tcaccgacga

2161  gccagtggcg ataagtcgtg tcttaccggg ttggactcaa gacgatagtt accggataag
      cggtcaccgc tattcagcac agaatggccc aacctgagtt ctgctatcaa tggcctattc

2221  gcgcagcggt cgggctgaac ggggggttcg tgcacacagc ccagcttgga gcgaacgacc
      cgcgtcgcca gcccgacttg ccccccaagc acgtgtgtcg ggtcgaacct cgcttgctgg

2281  tacaccgaac tgagatacct acagcgtgag ctatgagaaa gcgccacgct tcccgaaggg
      atgtggcttg actctatgga tgtcgcactc gatactcttt cgcggtgcga agggcttccc

2341  agaaaggcgg acaggtatcc ggtaagcggc agggtcggaa caggagagcg cacgagggag
      tctttccgcc tgtccatagg ccattcgccg tcccagcctt gtcctctcgc gtgctccctc

2401  cttccagggg gaaacgcctg gtatctttat agtcctgtcg ggtttcgcca cctctgactt
      gaaggtcccc ctttgcggac catagaaata tcaggacagc ccaaagcggt ggagactgaa

2461  gagcgtcgat ttttgtgatg ctcgtcaggg gggcggagcc tatggaaaaa cgccagcaac
      ctcgcagcta aaaacactac gagcagtccc cccgcctcgg ataccttttt gcggtcgttg

                                                  PciI
2521  gcggcctttt tacggttcct ggccttttgc tggccttttg ctcacatgtt ctttcctgcg
      cgccggaaaa atgccaagga ccggaaaacg accggaaaac gagtgtacaa gaaaggacgc

2581  ttatcccctg attctgtgga taaccgtatt accgcctttg agtgagctga taccgctcgc
      aatagggac taagacacct attggcataa tggcggaaac tcactcgact atggcgagcg

                                                     SapI
2641  cgcagccgaa cgaccgagcg cagcgagtca gtgagcgagg aagcggaaga gcgcctgatg
      gcgtcggctt gctggctcgc gtcgctcagt cactcgctcc ttcgccttct cgcggactac

2701  cggtattttc tccttacgca tctgtgcggt atttcacacc gcatatatgg tgcactctca
      gccataaaag aggaatgcgt agacacgcca taaagtgtgg cgtatatacc acgtgagagt

                                     BstZ17I
                                      AccI
2761  gtacaatctg ctctgatgcc gcatagttaa gccagtatac actccgctat cgctacgtga
      catgttagac gagactacgg cgtatcaatt cggtcatatg tgaggcgata gcgatgcact
```

```
              Tth111I
2821  ctgggtcatg gctgcgcccc gacacccgcc aacacccgct gacgcgccct gacgggcttg
      gacccagtac cgacgcgggg ctgtgggcgg ttgtgggcga ctgcgcggga ctgcccgaac

2881  tctgctcccg gcatccgctt acagacaagc tgtgaccgtc tccgggagct gcatgtgtca
      agacgagggc cgtaggcgaa tgtctgttcg acactggcag aggccctcga cgtacacagt

2941  gaggttttca ccgtcatcac cgaaacgcgc gaggcagctg cggtaaagct catcagcgtg
      ctccaaaagt ggcagtagtg gctttgcgcg ctccgtcgac gccatttcga gtagtcgcac

3001  gtcgtgaagc gattcacaga tgtctgcctg ttcatccgcg tccagctcgt tgagtttctc
      cagcacttcg ctaagtgtct acagacggac aagtaggcgc aggtcgagca actcaaagag

3061  cagaagcgtt aatgtctggc ttctgataaa gcgggccatg ttaagggcgg tttttcctg
      gtcttcgcaa ttacagaccg aagactattt cgcccggtac aattcccgcc aaaaaaggac

3121  tttggtcact gatgcctccg tgtaaggggg atttctgttc atgggggtaa tgataccgat
      aaaccagtga ctacggaggc acattccccc taaagacaag tacccccatt actatggcta

3181  gaaacgagag aggatgctca cgatacgggt tactgatgat gaacatgccc ggttactgga
      ctttgctctc tcctacgagt gctatgccca atgactacta cttgtacggg ccaatgacct

3241  acgttgtgag ggtaaacaac tggcggtatg gatgcggcgg gaccagagaa aaatcactca
      tgcaacactc ccatttgttg accgccatac ctacgccgcc ctggtctctt tttagtgagt

3301  gggtcaatgc cagcgcttcg ttaatacaga tgtaggtgtt ccacagggta gccagcagca
      cccagttacg gtcgcgaagc aattatgtct acatccacaa ggtgtcccat cggtcgtcgt

3361  tcctgcgatg cagatccgga acataatggt gcagggcgct gacttccgcg tttccagact
      aggacgctac gtctaggcct tgtattacca cgtcccgcga ctgaaggcgc aaaggtctga

                                                  Bpu10I
3421  ttacgaaaca cggaaaccga agaccattca tgttgttgct caggtcgcag acgttttgca
      aatgctttgt gcctttggct tctggtaagt acaacaacga gtccagcgtc tgcaaaacgt

3481  gcagcagtcg cttcacgttc gctcgcgtat cggtgattca ttctgctaac cagtaaggca
      cgtcgtcagc gaagtgcaag cgagcgcata gccactaagt aagacgattg gtcattccgt

                          PpuMI                         FspAI
3541  accccgccag cctagccggg tcctcaacga caggagcacg atcatgcgca cccgtggggc
      tggggcggtc ggatcggccc aggagttgct gtcctcgtgc tagtacgcgt gggcaccccg

              BglI
3601  cgccatgccg gcgataatgg cctgcttctc gccgaaacgt ttggtggcgg gaccagtgac
      gcggtacggc cgctattacc ggacgaagag cggctttgca aaccaccgcc ctggtcactg

3661  gaaggcttga gcgagggcgt gcaagattcc gaataccgca agcgacaggc cgatcatcgt
      cttccgaact cgctcccgca cgttctaagg cttatggcgt tcgctgtccg gctagtagca

3721  cgcgctccag cgaaagcggt cctcgccgaa aatgacccag agcgctgccg gcacctgtcc
      gcgcgaggtc gctttcgcca ggagcggctt ttactgggtc tcgcgacggc cgtggacagg

                                                    PshAI
3781  tacgagttgc atgataaaga agacagtcat aagtgcggcg acgatagtca tgccccgcgc
      atgctcaacg tactatttct tctgtcagta ttcacgccgc tgctatcagt acggggcgcg

3841  ccaccggaag gagctgactg ggttgaaggc tctcaagggc atcggtcgag atcccggtgc
      ggtggccttc ctcgactgac ccaacttccg agagttcccg tagccagctc tagggccacg

3901  ctaatgagtg agctaactta cattaattgc gttgcgctca ctgcccgctt tccagtcggg
      gattactcac tcgattgaat gtaattaacg caacgcgagt gacgggcgaa aggtcagccc
                                              <<.........lac..........<
                                                -   q  g  s   e  l  r

3961  aaacctgtcg tgccagctgc attaatgaat cggccaacgc gcggggagag gcggtttgcg
      tttggacagc acggtcgacg taattactta gccggttgcg cgcccctctc cgccaaacgc
      <............................lac..............................<
      s  v  q  r   a  l  q   m  l  s   d  a  l  a   r  p  s   a  t  q
```

```
4021  tattgggcgc cagggtggtt tttcttttca ccagtgagac gggcaacagc tgattgccct
      ataacccgcg gtcccaccaa aaagaaaagt ggtcactctg cccgttgtcg actaacggga
      <............................lac.............................<
      t  n  p  a   l  t  t   k  r  k   v  l  s  v   p  l  l   q  n  g

4081  tcaccgcctg gccctgagag agttgcagca agcggtccac gctggtttgc cccagcaggc
      agtggcggac cgggactctc tcaacgtcgt tcgccaggtg cgaccaaacg gggtcgtccg
      <............................lac.............................<
      k  v  a  q   g  q  s   l  q  l   l  r  d  v   s  t  q   g  l  l

                                  HpaI
4141  gaaaatcctg tttgatggtg gttaacggcg ggatataaca tgagctgtct tcggtatcgt
      cttttaggac aaactaccac caattgccgc cctatattgt actcgacaga agccatagca
      <............................lac.............................<
      r  f  d  q   k  i  t   t  l  p   p  i  y  c   s  s  d   e  t  d

                         EcoRV                                  BssHII
4201  cgtatcccac taccgagata tccgcaccaa cgcgcagccc ggactcggta atggcgcgca
      gcatagggtg atggctctat aggcgtggtt gcgcgtcggg cctgagccat taccgcgcgt
      <............................lac.............................<
      d  y  g  v   v  s  i   d  a  g   v  r  l  g   s  e  t   i  a  r

4261  ttgcgcccag cgccatctga tcgttggcaa ccagcatcgc agtgggaacg atgccctcat
      aacgcgggtc gcggtagact agcaaccgtt ggtcgtagcg tcacccttgc tacgggagta
      <............................lac.............................<
      m  a  g  l   a  m  q   d  n  a   v  l  m  a   t  p  v   i  g  e

4321  tcagcatttg catggtttgt tgaaaaccgg acatggcact ccagtcgcct tcccgttccg
      agtcgtaaac gtaccaaaca acttttggcc tgtaccgtga ggtcagcgga agggcaaggc
      <............................lac.............................<
      n  l  m  q   m  t  q   q  f  g   s  m  a  s   w  d  g   e  r  e

4381  ctatcggctg aatttgattg cgagtgagat atttatgcca gccagccaga cgcagacgcg
      gatagccgac ttaaactaac gctcactcta taaatacggt cggtcggtct gcgtctgcgc
      <............................lac.............................<
      a  i  p  q   i  q  n   r  t  l   y  k  h  w   g  a  l   r  l  r

                                                    BstEII
4441  ccgagacaga acttaatggg cccgctaaca gcgcgatttg ctggtgaccc aatgcgacca
      ggctctgtct tgaattaccc gggcgattgt cgcgctaaac gaccactggg ttacgctggt
      <............................lac.............................<
      a  s  v  s   s  l  p   g  a  l   l  a  i  q   q  h  g   l  a  v

4501  gatgctccac gcccagtcgc gtaccgtctt catgggagaa aataatactg ttgatgggtg
      ctacgaggtg cgggtcagcg catggcagaa gtaccctctt ttattatgac aactacccac
      <............................lac.............................<
      l  h  e  v   g  l  r   t  g  d   e  h  s  f   i  i  s   n  i  p

4561  tctggtcaga gacatcaaga aataacgccg gaacattagt gcaggcagct tccacagcaa
      agaccagtct ctgtagttct ttattgcggc cttgtaatca cgtccgtcga aggtgtcgtt
      <............................lac.............................<
      t  q  d  s   v  d  l   f  l  a   p  v  n  t   c  a  a   e  v  a

                                        BclI          MluI
4621  tggcatcctg gtcatccagc ggatagttaa tgatcagccc actgacgcgt tgcgcgagaa
      accgtaggac cagtaggtcg cctatcaatt actagtcggg tgactgcgca acgcgctctt
      <............................lac.............................<
      i  a  d  q   d  d  l   p  y  n   i  i  l  g   s  v  r   q  a  l

4681  gattgtgcac cgccgcttta caggcttcga cgccgcttcg ttctaccatc gacaccacca
      ctaacacgtg gcggcgaaat gtccgaagct gcggcgaagc aagatggtag ctgtggtggt
      <............................lac.............................<
      l  n  h  v   a  a  k   c  a  e   v  g  s  r   e  v  m   s  v  v

4741  cgctggcacc cagttgatcg gcgcgagatt taatcgccgc gacaatttgc gacggcgcgt
      gcgaccgtgg gtcaactagc cgcgctctaa attagcggcg ctgttaaacg ctgccgcgca
      <............................lac.............................<
      v  s  a  g   l  q  d   a  r  s   k  i  a  a   v  i  q   s  p  a
```

```
4801  gcagggccag actggaggtg gcaacgccaa tcagcaacga ctgtttgccc gccagttgtt
      cgtcccggtc tgacctccac cgttgcggtt agtcgttgct gacaaacggg cggtcaacaa
      <.........................lac..........................<
      h  l  a  l   s  s  t   a  v  g   i  l  l  s   q  k  g   a  l  q

4861  gtgccacgcg gttgggaatg taattcagct ccgccatcgc cgcttccact ttttcccgcg
      cacggtgcgc caacccttac attaagtcga ggcggtagcg gcgaaggtga aaaagggcgc
      <..............lac...............<<
      q  a  v  r   n  p  i   y  n  l   e  a  m

4921  ttttcgcaga aacgtggctg gcctggttca ccacgcggga aacggtctga taagagacac
      aaaagcgtct ttgcaccgac cggaccaagt ggtgcgccct ttgccagact attctctgtg

           BstAPI
4981  cggcatactc tgcgacatcg tataacgtta ctggtttcac attcaccacc ctgaattgac
      gccgtatgag acgctgtagc atattgcaat gaccaaagtg taagtggtgg gacttaactg

                                                       PflMI
5041  tctcttccgg gcgctatcat gccataccgc gaaaggtttt gcgccattcg atggtgtccg
      agagaaggcc cgcgatagta cggtatggcg ctttccaaaa cgcggtaagc taccacaggc

                                        EcoNI
5101  ggatctcgac gctctccctt atgcgactcc tgcattagga agcagcccag tagtaggttg
      cctagagctg cgagagggaa tacgctgagg acgtaatcct tcgtcgggtc atcatccaac

                                           SphI
5161  aggccgttga gcaccgccgc cgcaaggaat ggtgcatgca aggagatggc gcccaacagt
      tccggcaact cgtggcggcg gcgttcctta ccacgtacgt tcctctaccg cgggttgtca

5221  cccccggcca cggggcctgc caccataccc acgccgaaac aagcgctcat gagcccgaag
      gggggccggt gccccggacg gtggtatggg tgcggctttg ttcgcgagta ctcgggcttc

5281  tggcgagccc gatcttcccc atcggtgatg tcggcgatat aggcgccagc aaccgcacct
      accgctcggg ctagaagggg tagccactac agccgctata tccgcggtcg ttggcgtgga

           SgrAI                                           BglII
5341  gtggcgccgg tgatgccggc cacgatgcgt ccggcgtaga ggatcgagat ctcgatcccg
      caccgcggcc actacggccg gtgctacgca ggccgcatct cctagctcta gagctagggc

5401  cgaaat
      gcttta
```
